# Aggregation-induced emission and aggregation-promoted photochromism of bis(diphenylmethylene)dihydroacenes†
†Electronic supplementary information (ESI) available: Details of the synthesis and characterization. Crystallographic information files (CIF), ^1^H NMR, ^13^C NMR, HRMS, UV and PL spectra, and details of the DFT calculations. CCDC 1024629–1024631 and 1036099. For ESI and crystallographic data in CIF or other electronic format see DOI: 10.1039/c5sc00900f


**DOI:** 10.1039/c5sc00900f

**Published:** 2015-04-03

**Authors:** Zikai He, Liang Shan, Ju Mei, Hong Wang, Jacky W. Y. Lam, Herman H. Y. Sung, Ian D. Williams, Xiao Gu, Qian Miao, Ben Zhong Tang

**Affiliations:** a HKUST Shenzhen Research Institute , No. 9 Yuexing 1st RD, South Area, Hi-tech Park Nanshan , Shenzhen 518057 , China . Email: tangbenz@ust.hk; b Department of Chemistry , The Hong Kong University of Science and Technology (HKUST) , Clear Water Bay , Kowloon , Hong Kong , China; c Department of Chemistry , The Chinese University of Hong Kong , Shatin, New Territories , Hong Kong , China . Email: miaoqian@cuhk.edu.hk; d Guangdong Innovative Research Team , SCUT-HKUST Joint Research Laboratory , State Key Laboratory of Luminescent Materials and Devices , South China University of Technology (SCUT) , Guangzhou 510640 , China

## Abstract

Reported herein is a new class of pure polycyclic hydrocarbon molecules, designed through a novel aggregation-induced emission (AIE) strategy, with unexpected photochromic properties. The restriction of intramolecular motion was found as a comprehensive mechanism for the AIE effect. The photochromism mechanism study revealed that the photocyclization reaction of *cis*-stilbene, the molecular conformation in the single crystal and the tetracene backbone should contribute to the unique photo behavior. In particular, the fast responsive, photo-reversible and thermo-irreversible photochromic effect facilitated in the solid state opens a new field of aggregation-promoted photochromism.

## Abstract 2

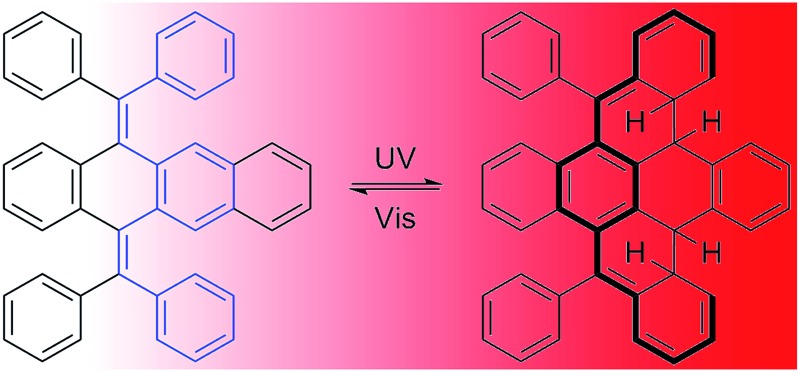
Solid-state photochromism was found in bis(diphenylmethylene)dihydrotetracene, caused by photocyclization of the embedded *cis*-stilbene motifs.

## Introduction

**Fig. 1:**
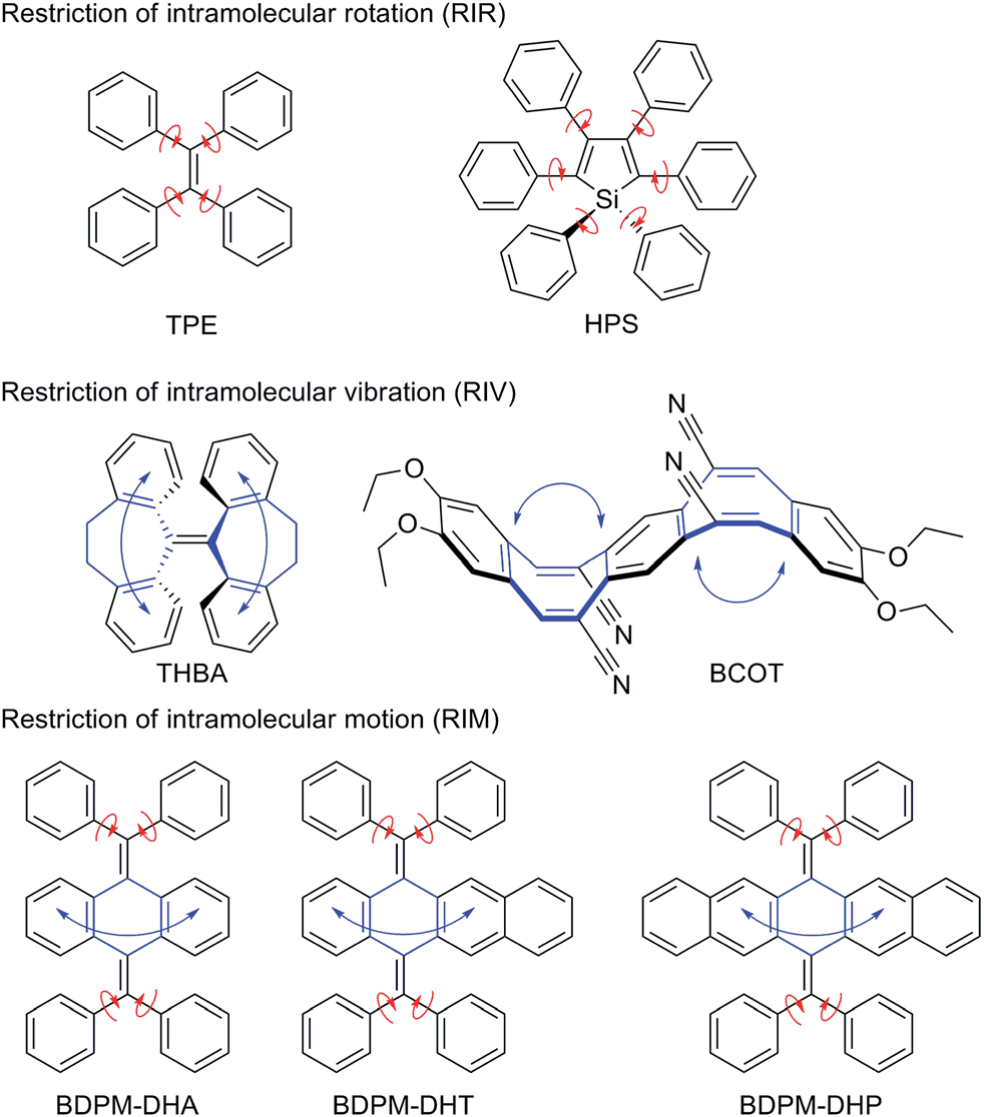
AIE mechanism hypothesis (red arrow: rotation; blue arrow: vibration) and structures of the bis(diphenylmethylene)-dihydroacenes.

Aggregation-induced emission (AIE) refers to the phenomenon that a class of luminogens are molecularly nonemissive but become highly emissive when clustered into aggregates. Luminogenic materials with AIE characteristics have attracted extensive interest and found varied applications.^1,2^ Based on two archetypal AIE luminogens, tetraphenylethene (TPE) and hexaphenylsilole (HPS), which contain multiple phenyl peripheries (rotor) linked to the ethylene or silole core (stator) *via* single bonds (axis), the restriction of intramolecular rotation (RIR) process was proposed as the main cause of the AIE effect (Fig. 1).^3^ This RIR mechanism has been successfully utilized to explore a large variety of new fluorescent and phosphorescent materials with high luminescence quantum yields in their solid states. However, some newly emerging AIE systems, such as the nonplanar THBA^4^ and BCOT^5^ (Fig. 1), have an absence of multiple rotors. As is well known, rotation and vibration are the two main modes of molecular motions accompanied by energy consumption. We proposed that the AIE effect of THBA and BCOT may mainly originate from the restriction of intramolecular vibrations.^6^ The phenyl rings could be viewed as vibrational parts which are connected by a flexible bridge, heptagon for THBA and octagon for BCOT. Upon aggregation, the substantial intramolecular vibrations are restricted to block the nonradiative decay pathway and open the radiative decay pathway. We integrated the RIR with the restriction of intramolecular vibration, as the restriction of intramolecular motion (RIM), which will be of great importance for extending the AIE scope.^6^

Accordingly, 9,10-bis(diphenylmethylene)-9,10-dihydroanthracene (BDPM-DHA), 5,12-bis(diphenylmethylene)-5,12-dihydrotetracene (BDPM-DHT) and 6,13-bis(diphenylmethylene)-6,13-dihydropentacene (BDPM-DHP) were designed, which contain multiple phenyl groups as the rotational parts and dihydroacenes as the vibrational parts (Fig. 1). All of the three molecules are AIE active, which is in support of our RIM mechanism hypothesis. Furthermore, an unprecedented photochromic effect was found in the BDPM-DHT crystals. The colorless BDPM-DHT crystals turned red and their blue emission disappeared upon UV irradiation, which can be gradually recovered *via* room light treatment.

Photochromic materials have been attracting increasing interest due to their great importance in fundamental research and practical applications.^7^ A large number of photochromic systems, including azobenzene,^8^ spiropyran,^9^ diarylethene,^10^ *etc.*, have been reported and exhibited good performances in photochemical, biological and nanotechnological applications. However, these systems always have heteroatoms (*e.g.*, N, S, and O), resulting in complicated synthesis, high cost and therefore limited practical applications. Few photochromic systems in the literature are based on pure hydrocarbon compounds, due to their thermal instability. The exploration of novel photochromic systems and the understanding of their working mechanisms are drawing continuous attention.^11^ Particularly, the development of facilely synthesized novel photochromic systems is highly challenging and in demand.^12^ Reported herein is a novel aggregation-promoted photochromic system based on a pure polycyclic hydrocarbon compound. This system is facilely synthesized and exhibits good thermal stability as well as a fast photo-response.

## Results and discussion

### Synthesis, crystal structures and aggregation-induced emission

From commercially available acenequinones, BDPM-DHA, BDPM-DHT and BDPM-DHP were synthesized according to the literature method with modifications.^13^ Following typical Corey–Fuchs and Suzuki–Miyaura reactions, grams of the final products were obtained, in high yield, which were purified by chromatography and recrystallization from chloroform–hexane solution (Scheme S1†). Although the syntheses of BDPM-DHA and BDPM-DHP have been reported in earlier literature,^13,14^ their AIE properties and crystal structures are investigated here for the first time.

The photophysical properties of BDPM-DHA and BDPM-DHP were investigated both in their solution and aggregate states. It was verified that the compounds behave as typical AIE compounds, with no emission in their dilute THF solution and a fluorescence emission, at 455 nm and 530 nm, respectively, in their aggregate states (Fig. S4 and S5†). Because BDPM-DHP possesses morphology-dependent luminescence properties,^15^ its emission peak red shifts with increasing water fraction. The solid quantum yields of BDPM-DHA and BDPM-DHP are 15% and 4%, respectively (excited at 325 nm). The AIE properties of BDPM-DHA and BDPM-DHP should originate from the effective RIM process. The excited states in solution decay through the nonradiative pathways because of the intramolecular motions such as phenyl group rotation and acene backbone vibration. Upon aggregation, these intramolecular motions are restricted. The radiative decay pathway turns on.

**Fig. 2:**
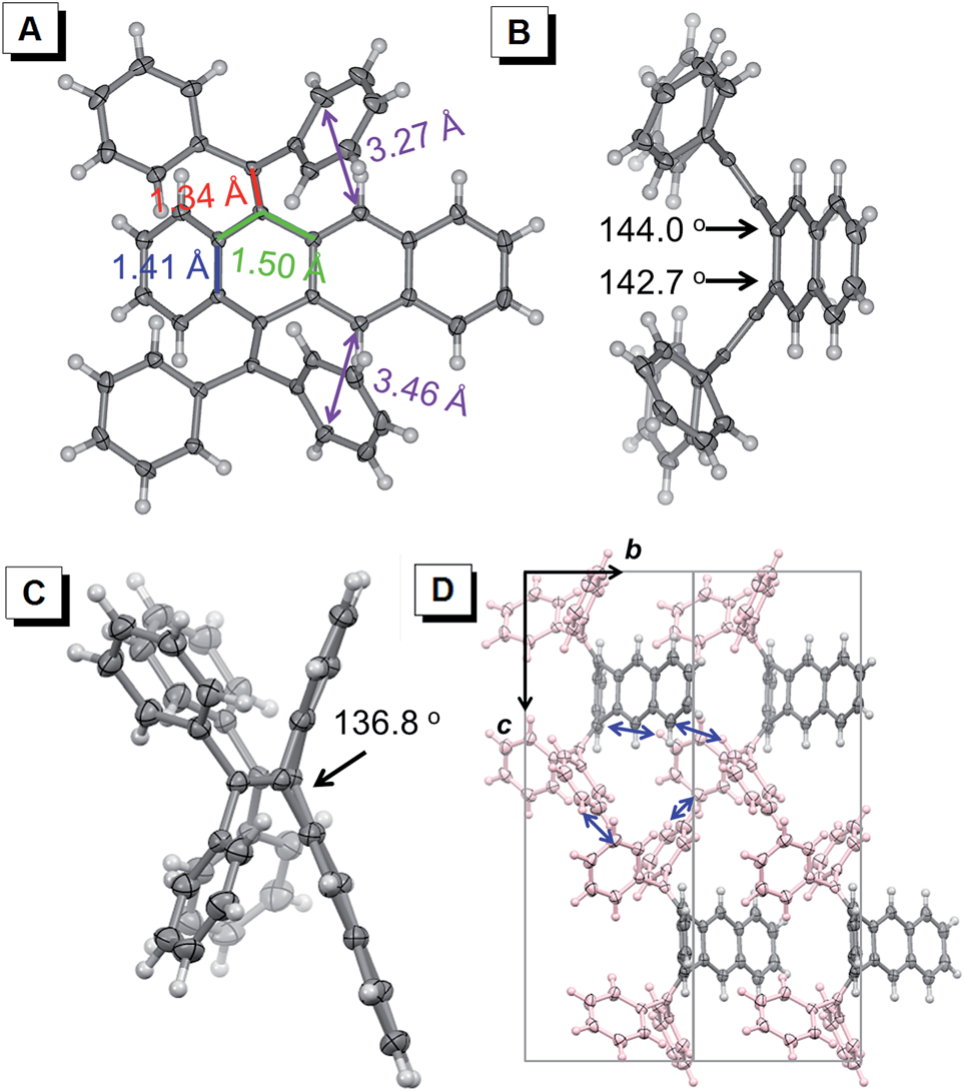
(A) Top view and (B and C) side views of BDPM-DHT with the carbon atom positions shown as 50% probability ellipsoids. (D) Molecular packing of BDPM-DHT (solvent free) as viewed along the *a* axis of the unit cell, with diphenylmethylene groups shown in pink (carbon and hydrogen atoms are shown in gray and white, respectively).

Single crystals of BDPM-DHA, BDPM-DHT and BDPM-DHP suitable for X-ray crystallography were grown by slowly diffusing *n*-hexane into CHCl_3_ solution or solvent evaporation.^16^ Shown in Fig. 2 are the crystal structures of BDPM-DHT (grown by slow evaporation of a THF solution). As highlighted in Fig. 2A, the C–C bonds shown in red, green and blue have bond lengths close to a typical C–C double bond, single bond and benzene double bond, respectively. These bond lengths indicate that the diphenylmethyl groups have weak conjugation with the tetracene backbone, in agreement with its highly contorted structure. Viewed from the side, the BDPM-DHT molecule bends with large dihedral angles for both the diphenylmethylene groups (Fig. 2B) and tetracene backbone (Fig. 2C). This nonplanar structure can be attributed to the steric repulsion between these groups. The BDPM-DHA and BDPM-DHP in the crystals adopt similar distorted structures, as summarized in the ESI (Fig. S1 and S2†). Shown in Fig. 2D is the molecular packing of BDPM-DHT, which is built through weak C–H···C (blue arrow) interactions. As a result, the BDPM-DHT molecules are locked and separated in the crystal. The BDPM-DHT crystals become emissive because the nonradiative decay pathways, such as intramolecular rotations and vibrations are blocked in the crystals.

### Aggregation-promoted photochromism

**Fig. 3:**
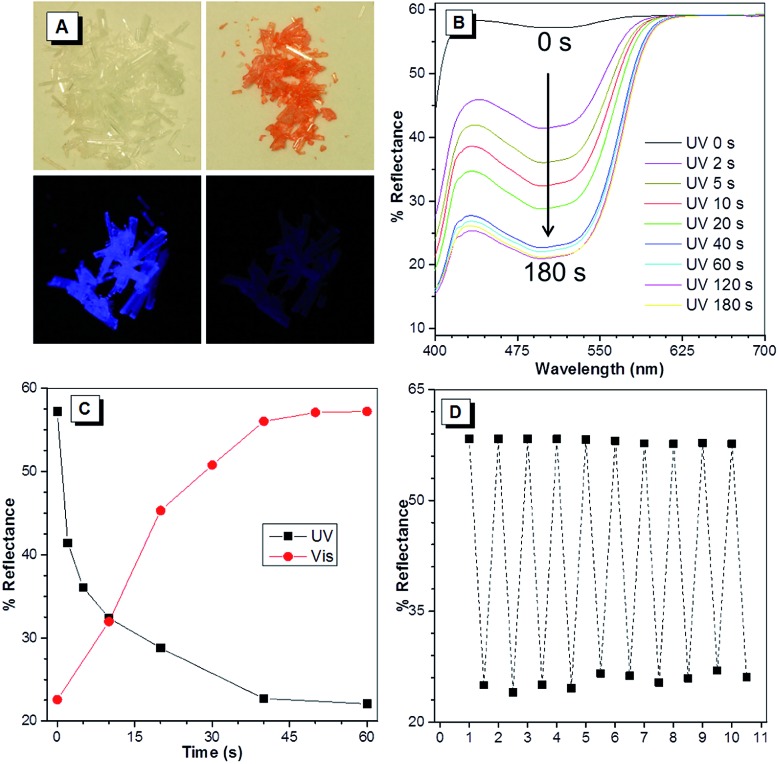
(A) Top: room light images of the BDPM-DHT crystals before (left) and after (right) UV irradiation; bottom: UV light images of the BDPM-DHT crystals before (left) and after (right) UV irradiation. (B) Changes in the UV-vis reflectance spectra of the BDPM-DHT crystals upon irradiation at *λ* = 365 nm. (C) The plot of the reflectance change at *λ* = 498 nm as a function of UV irradiation or visible light exposure time. (D) Fatigue resistance of crystals of BDPM-DHT upon irradiating at 365 nm (1 minute) and standing in room light (1 minute) alternately (the reflectance was measured at 498 nm).

As shown in Fig. 3A, the colorless needle-shaped crystals of BDPM-DHT exhibit blue emission under UV lamp irradiation (365 nm) in accordance with their AIE characteristics. It was unexpectedly found that the colorless crystals became red when the UV lamp was removed, even after a very short irradiation time (∼1 second). Meanwhile, the blue emission significantly faded. When placed under room light, the red crystals gradually returned to colorless, accompanied with a blue emission recovery. Therefore, BDPM-DHT crystals show a typical photochromic effect. Meanwhile, the photochromic reaction itself could be considered as the quenching process of the fluorescence. As shown in Fig. S8,† the PL intensity of the BDPM-DHA and BDPM-DHP films remained unchanged, indicating that they do not show such a photochromic effect.

To further investigate the color change of the red crystals, they were treated under different conditions. When placed in darkness at room temperature for 2 days, the red crystals remained unchanged, indicating that they were thermally irreversible, which was indispensable for application as optical memories and switches for a photochromic system. When placed at an elevated temperature of about 60 °C, before the crystals collapse (Fig. S9†), the red crystals can survive for several hours in darkness. X-ray crystallographic analysis of the red crystals was also performed under dark conditions with continuous UV irradiation (Fig. S10†), and the results revealed that they were identical to the colorless ones, suggesting that the photochromic reaction occurred in a very small amount. After cracking the red crystals, the colorless inner part told us that the photochromism occurred only on the crystal surface. On further treating the cracked crystals with UV light, the colorless inner part turned red, as the original crystal did, verifying that the photochromism was not induced by impurities on the crystal surface. On prolonging the UV irradiation time to 60 minutes, the inner part remains unreacted, which may be attributed to the UV absorption of the red crystal surface.

The UV response of BDPM-DHT in its solution state was then tested in 10^–5^ M THF and CHCl_3_ solutions, monitored by UV-vis absorption. However, no detectable change was observed upon UV irradiation for 5–10 minutes (Fig. S6†). Just like in AIE, the aggregation plays an important role in the photochromic process. In the solution, the excited molecules can easily nonradiatively decay through intramolecular motions and solvent molecule collisions. Upon aggregation, these pathways are blocked and the blue emission followed by the photochromic reaction were facilitated. As a result, it is an aggregation-promoted photochromism system. To exclude a solvate effect of CHCl_3_, solvent-free crystals were successfully obtained and investigated for comparison. As expected, they show a similar photochromic behavior under UV and room light irradiation, with a color change between colorless and yellow. The slight difference in the color change is due to an even smaller amount of surface reaction.

The photochromic processes of the BDPM-DHT crystals were then investigated by UV-vis reflectance spectroscopy. Before UV irradiation, the colorless crystal exhibits a maximum absorption at 370 nm, which suggests weak conjugation in the molecules. However, upon irradiation at 365 nm, a strong absorption band peaked at 498 nm emerges and increases with the progress of the photo-irradiation (Fig. 3B). The absorbance reaches half-peak width within 5 seconds and saturates after 60 seconds. Additionally, the progress of the room light response was also recorded using the same method (Fig. S7†). The increase and decrease of the reflectance at 498 nm as a function of time were plotted and used to monitor the photo response processes. As shown in Fig. 3C, the crystals responded rapidly in the first 10 seconds and almost finished responding within 60 seconds, towards UV and visible light. As shown in Fig. 3D, the BDPM-DHT crystals were repeatedly switched between the red and colorless states 10 times and the reflectance at 498 nm stayed almost constant, without any apparent degradation, indicating their good fatigue resistance.

### Photochromic mechanism

**Fig. 4:**
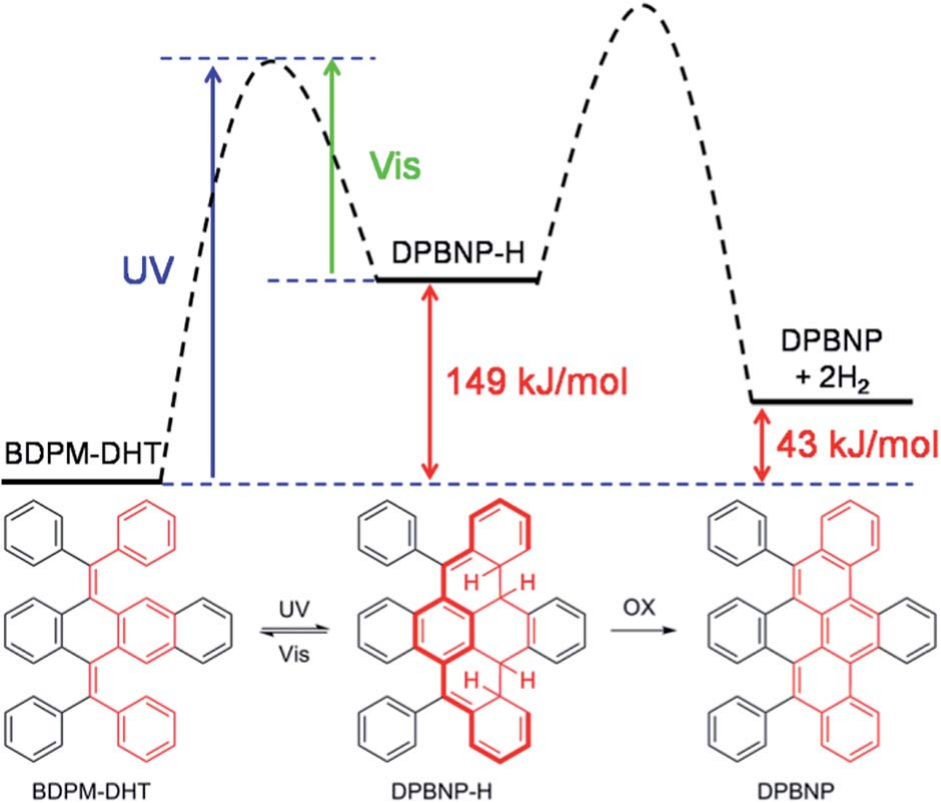
Proposed mechanism of the photochromic process and the calculated reaction coordinate diagram.

Due to the presence of the two fixed *cis*-stilbene motifs and the short distance between the phenyl ring and the tetracene backbone (Fig. 2A), a mechanism for the color change of the BDPM-DHT crystals upon UV irradiation was proposed as depicted in Fig. 4. Here, the photochromic effect of BDPM-DHT is ascribed to photo-induced ring closing from BDPM-DHT to DPBNP-H, which has a larger π conjugation as highlighted by the bold-red line. DFT calculations, further crystal structure analysis and photo oxidation reactions were applied to verify the photochromism hypothesis and investigate the DPBNP-H intermediate.

On going from BDPM-DHT to DPBNP-H, three benzene rings are broken. However, after DFT calculations, we found that the energy difference between BDPM-DHT and DPBNP-H is merely about 149 kJ mol^–1^, which is close to the resonance energy of only one benzene ring. The newly formed π-conjugation was thought to make a big contribution to the stabilization of the intermediate DPBNP-H. The possibility of photocyclization on the benzene-ring side rather than on the naphthalene-ring side was partially excluded, because of an even larger energy difference between BDPM-DHT and DPBNP-H′ of 172.6 kJ mol^–1^ (Fig. S13†).

Meanwhile, the same calculations were performed on the BDPM-DHA and BDPM-DHP systems. The energy difference increases to 158 kJ mol^–1^ for BDPM-DHA and 164 kJ mol^–1^ for BDPM-DHP. Although the calculation results are in agreement with no photochromic behavior (see Fig. S11†), the energy differences among the three systems are relatively small. We further studied their molecular and crystal structural differences. Among the three systems, BDPM-DHT has the highest similarity between its backbone and that of DPBNP-H. For BDPM-DHT, the aromatic parts of the tetracene backbone have almost no change before and after the cyclization, remaining as one naphthalene ring and one benzene ring. However, for BDPM-DHA, the aromatic parts of the anthracene backbone change from two benzene rings to one naphthalene ring. For BDPM-DHP, the aromatic parts of the pentacene backbone change from two naphthalene rings to one anthracene ring and one benzene ring. According to Clar's rule, such changes are disfavored in energy. As summarized in Table S2,† although all of the distances between the possible photoactive carbon atoms are less than the 4.2 Å required for solid photocyclization,^17^ the BDPM-DHT (solvent-free and CHCl_3_) molecules in the single crystals have shorter distance pairs than BDPM-DHA and BDPM-DHP, indicating the highest possibility of electronic cyclization reaction and exhibited by their photochromic UV irradiation responses.

In the presence of air, the dihydrophenanthrene can be oxidized to phenanthrene irreversibly. To further verify the mechanism, we also tried to obtain oxidation product DPBNP from the intermediate DPBNP-H as an indirect proof. Firstly, we extended the UV irradiation time to 2 days in air. The BDPM-DHT crystal surface irreversibly turned yellow and a peak related to DPBNP appeared in the high-resolution mass spectrum (Fig. S14†). In contrast, the BDPM-DHT crystal was successfully recovered to colorless after irradiation in a N_2_ atmosphere for 24 hours, without oxidation. Secondly, we used a Katz-modified Mallory photocyclization method to treat BDPM-DHT.^18^ DPBNP was obtained in a high yield. To our surprise, the reaction was completed in 20 minutes, which is much faster than that of BDPM-DHA and BDPM-DHP.^19^ The photochromic effect of BDPM-DHT should help with the formation of the dihydro intermediate and accelerate the reaction. The DPBNP structure tells us that the photocyclization reaction occurred at the naphthalene-ring part. It is worth noting that the DPBNP is an aggregation-induced quenching molecule, which is the opposite to its precursor BDPM-DHT (AIE). The partial locking of the phenyl rings and large π-plane formation should account for this property.

Through controlled irradiation and photocyclization product analysis, the dihydro intermediate was believed to be the most probable photochromic product *via* a 6-π electronic cyclization reaction. The theoretical calculations also prove the proposed mechanism. Due to its small amount and the fast response towards room light, continuous efforts were made to find DPBNP-H but failed. It is well known that *cis*-stilbene can undergo a photocyclization reaction to yield dihydrophenanthrene.^20^ However, dihydrophenanthrene is highly unstable and returns to stilbene very quickly even in darkness,^21^ which creates an obstacle for the characterization of the dihydro-intermediate (*e.g.*, DPBNP-H in our system). Therefore, the phenyl rings of stilbene are usually replaced by heteroaryl rings, such as thiophene, furan and pyrrole, to prolong the lifetime of the dihydro intermediates, because heteroaryl rings have smaller aromaticity.^21^ Therefore, 5,12-bis(dithiophenylmethylene)-5,12-dihydrotetracene (BDTM-DHT) was chosen for further verification of the mechanism. The backbone is the same as for BDPM-DHT, but four thiophenyl groups were used to replace the four phenyl groups. The amount of photochromic reaction in BDTM-DHT was enhanced due to the smaller aromaticity of the thiophene ring. The photocyclization mechanism would be directly proven by the existence of cyclized-BDTM-DHT (c-BDTM-DHT). As predicted (Fig. S15†), upon photocyclization, signals at ∼4 ppm relating to alkyl protons and a signal at ∼6.5 ppm relating to vinylic protons appear. As shown in Fig. S16 and S17,† after UV irradiation, signals at 4.4 ppm, 4.8 ppm, and 6.4 ppm were observed in both CDCl_3_ and CD_2_Cl_2_ solution. Although they are poorly resolved, these new signals still indicate the existence of c-BDTM-DHT. The differences from the predicted spectra may arise from chirality and asymmetry of the c-BDTM-DHT structure or a one-sided photocyclization product. The weak intensity of the signals suggests a low quantum yield of our photochromic system.

**Fig. 5:**
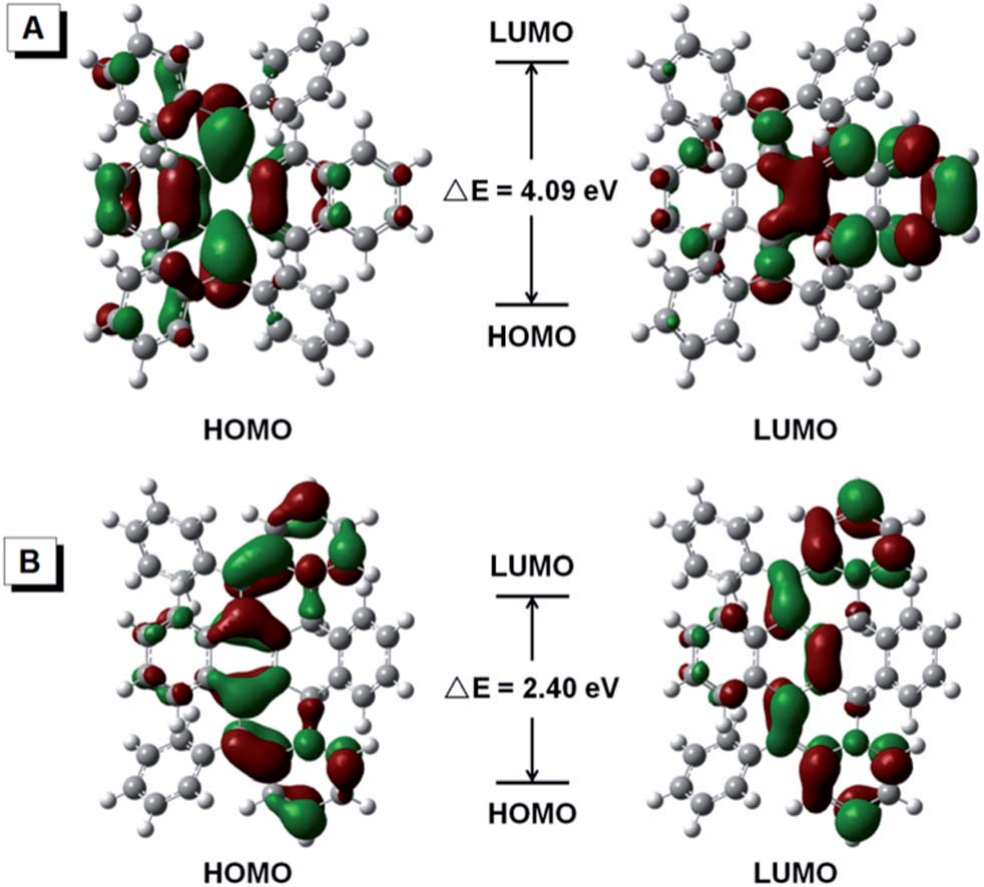
Molecular orbital amplitude plots (isovalue = 0.03) of the HOMO and LUMO energy levels of BDPM-DHT (A, open form) and DPBNP-H (B, closed form).

As shown in Fig. 5, the HOMO and LUMO energy levels of BDPM-DHT were calculated and found localized at the dimethylene-cyclohexadiene part and the naphthalene part, respectively, proving its weak intramolecular conjugation and distorted structure. For DPBNP-H, both the HOMO and LUMO are delocalized throughout the newly formed π-bonds. Meanwhile, the energy gap between the HOMO and LUMO is dramatically decreased from 4.09 eV to 2.40 eV, which is in good agreement with its optical energy gap and the color change from colorless to red.

## Conclusions

In summary, this study puts forth a new photochromic system based on an AIE luminogen, which is composed of a pure polycyclic hydrocarbon compound without any heteroatoms. Grams of BDPM-DHT were successfully synthesized through simple reactions. In particular, fast responsive, photo-reversible and thermo-irreversible photochromic properties were realized in the solid state, which are crucial for practical application. A photocyclization reaction was proposed as the mechanism for the photochromic process, whose energy barrier is partially counterbalanced by newly formed π-conjugation and the tetracene backbone. Similar to AIE, the solid-state photochromic response makes BDPM-DHT the first aggregation-promoted photochromic system, which is facilitated by RIM and functions as a novel photochromic system.
