# Supplementary material for: Aggregation-induced emission and aggregation-promoted photochromism of bis(diphenylmethylene)dihydroacenes
Source: Chem Sci. 2015 Apr 3;6(6):3538–43. doi: 10.1039/c5sc00900f (PMC5500900; doi:10.1039/c5sc00900f)
Supplement: Supplementary file 1 [file SC-006-C5SC00900F-s001.pdf]

Electronic Supplementary Material (ESI) for Chemical Science.
This journal is © The Royal Society of Chemistry 2015

Supporting Information for:

Aggregation-Induced Emission and Aggregation-Promoted Photochromism of Bis(diphenylmethylene)dihydroacenes

Zikai He,[ab] Liang Shan,[c] Ju Mei,[ab] Hong Wang,[ab] Jacky W. Y. Lam,[ab] Herman H. Y. Sung,[b] Ian D. Williams,[b] Xiao Gu,[c] Qian Miao*[c] and Ben Zhong Tang*[abd]

[a] HKUST Shenzhen Research Institute, No. 9 Yuexing 1st RD, South Area, Hi-tech Park Nanshan, Shenzhen 518057, China. E-mail: tangbenz@ust.hk
[b] Department of Chemistry, The Hong Kong University of Science and Technology (HKUST) Clear Water Bay, Kowloon, Hong Kong, China.
[c] Department of Chemistry, the Chinese University of Hong Kong, Shatin, New Territories, Hong Kong, China. E-mail: miaoqian@cuhk.edu.hk
[d] Guangdong Innovative Researh Team, SCUT-HKUST Joint Research Laboratory, State Key Laboratory of Luminescent Materials and Devices, South China University of Technology (SCUT), Guangzhou 510640, China.

Table of Contents

1. Synthesis
2. Single crystal structures
3. Aggregation-induced emission properties of BDPM-DHA and BDPM-DHP
4. UV-vis absorption in solution
5. UV-vis reflectance absorption
6. Differential scanning calorimetry and thermal gravimetric analysis
7. Instruments for *in-situ* single crystal X-ray analysis
8. Photocyclization of *cis*-stillbene
9. Density functional theory calculation
10. High resolution mass spectrum
11. ^{1}H-NMR analysis of photocyclization mechanism
12. Electronic structures summary
13. NMR spectra

General: Tetrahydrofuran and toluene were distilled from sodium benzophenone ketyl under nitrogen immediately prior to use. All the chemicals and other regents employed were commercially available and used without any further purification if not specified elsewhere. ^{1}H-NMR and ^{13}C-NMR spectra recorded on a Brucker ADVANCE III 400MHz spectrometer in deuterated chloroform using tetramethylsilane (TMS; δ = 0) as internal reference. UV-vis solution spectra were measured on a Milton Roy Spectronic 3000 Array spectrophotometer. UV-vis reflectance spectra were measured on Perkin Elmer UV-vis Lambda 20 spectrometer. Photoluminescence was recorded on a Perkin-Elmer LS 55 spectrofluorometer. The MALDI-TOF mass spectrum was recorded on a GCT premier CAB048 mass spectrometer. Differential Scanning Calorimetry (DSC) was performed on Perkin Elmer Differential Scanning Calorimeter Pyris 1.

1. Synthesis

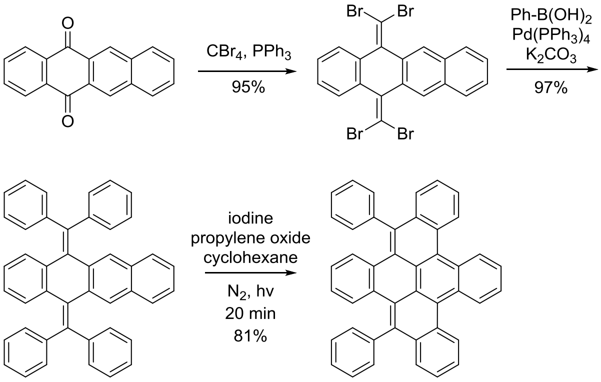

Scheme S1 Synthesis of 5,12-bis(diphenylmethylene)-5,12-dihydrotetracene (**BDPM-DHT**) and photocyclization conversion.

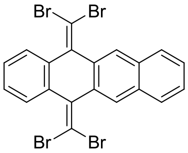

5, 12-bis(dibromomethylene)-5,12-dihydrotetracene

A mixture of CBr_4 (8.6 g, 25.9 mmol) and PPh_3 (13.6 g, 51.8 mmol) in 100 ml dry toluene was stirred for 30 minutes at room temperature before 5,12-tetracenequinone (2.06 g, 8.0 mmol) was added in one portion and the solution was heated to reflux for 24 h. The reaction mixture was cooled to room temperature; the solid was removed by filtration and washed with toluene. The filtrates were concentrated under reduced pressure and the crude product was obtained. The crude product was triturated with ethanol and filtered to give pure product (4.3 g) in 95% yield.

^{1}H-NMR(CDCl$_3$) δ (ppm): 8.26 (s, 2H), 7.86(m, 4H), 7.3 (dd, J_1=6.4 Hz, J_2=3.2 Hz, 2H), 7.31 (dd, J_1=5.6 Hz, J_2=3.2 Hz, 2H). ^{13}C-NMR(CDCl$_3$) δ (ppm): 90.59, 127.25, 127.33, 127.44, 127.93, 128.29, 131.79, 133.36, 136.17, 139.73. MALDI-TOF mass: m/z = 569.7479 (cacld. 569.7475).

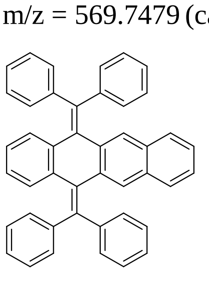

5, 12-bis(diphenylmethylene)-5,12-dihydrotetracene (BDPM-DHT)

To a solution of 5,12-bis(dibromomethylene)-5,12-dihydrotetracene (2.26 g, 4.0 mmol) and phenyl boronic acid (3.90 g, 32 mmol) in 100 mL toluene was added 5 ml ethanol and 5 ml water. The mixture was stirred at room temperature and bubbled with nitrogen for 30 minutes. Then K_2CO_3 (5.52 g, 40 mmol) and $Pd(PPh_3)_4$ (0.23 g, 0.20 mmol) were added in one portion and the mixture was heated to reflux for 24 hours. The reaction mixture was cooled to room temperature and filtered through a pad (silicon gel height: 5 cm), washing with dichloromethane until the filtrate was clean. The filtrates were concentrated under reduced pressure and the crude product was obtained. The crude product was then triturated with ethanol and filtered to give product (2.18 g) in 97% yield. Firstly, run fast chromatography using CH_2Cl_2:hexane = 1: 4 as eluent; then dissolve in $CHCl_3$ and put them in the hexane atmosphere, high purity products are obtained as colorless crystals after 2 days.

^{1}H-NMR($CDCl_3$) δ (ppm): 7.47 (m, 10H), 7.35 (dd, J_1=6.4 Hz, J_2=3.2 Hz, 2H), 7.31-7.13 (m, 14H), 7.03 (dd, J_1=6.0 Hz, J_2=3.6 Hz, 2H), 6.75 (dd, J_1=6.0 Hz, J_2=3.2 Hz, 4H). ^{13}C-NMR($CDCl_3$) δ (ppm): 125.52, 126.71, 126.76, 126.98, 127.63, 128.13, 128.26, 128.38, 129.69, 129.78, 131.28, 135.64, 135.74, 138.02, 140.41, 142.44, 142.46. MALDI-TOF mass: m/z = 558.2359 (cacld. 558.2348).

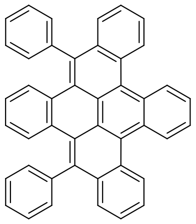

13,18-diphenylbenzo[*h*]naphtho[*1,2,3,4-rst*]pentaphene(DPBNP)

A standard photochemical reaction vessel with a double-walled quartz immersion well was used for the photo coupling, and a circulating chiller was used to provide cooling water to the quartz immersion well. N_2 was bubbled through a stirred solution of 5, 12-bis(diphenylmethylene)-5,12-dihydrotetracene (260mg, 0.50 mmol)， iodine (0.64 g, 2.50 mmol), and propylene oxide (20 mL) in 300 ml cyclohexane for 20 minutes before photo-irradiation, and the N_2 flow was maintained throughout irradiation with a 500 W high-pressure mercury vapor lamp placed in the immersion quartz well. The progress of the reaction was monitored by TLC. After completion of the reaction (20 minutes), the solvent was evaporated under reduced pressure. 225 mg 13,18-diphenylbenzo[h]naphtho[1,2,3,4-rst]- pentaphene was obtained as yellow powder in a yield of 81% after column chromatography.

^{1}H-NMR(CD_2Cl_2) δ(ppm): 9.17 (d, J=8.4Hz, 2H), 8.97 (dd, J_1=6.4 Hz, J_2=3.2 Hz, 2H), 7.91(dd, J_1=4.4 Hz, J_2=0.8 Hz, 2H), 7.78 (dd, J_1=6.4 Hz, J_2=3.2 Hz, 2H), 7.71-7.50 (m, 14H), 7.38 (dd, J_1=6.4 Hz, J_2=3.2 Hz, 2H), 6.80 (dd, J_1=6.4 Hz, J_2=3.2 Hz, 2H). ^{13}C-NMR($CDCl_3$) δ(ppm): 124.30, 125.61, 125.83, 126.20, 126.27, 126.49, 127.65, 127.79, 127.89, 128.83, 129.24, 129.89, 130.49, 132.23, 133.62, 135.71, 141.61. MALDI-TOF mass: m/z = 554.2036 (cacld. 554.2035).

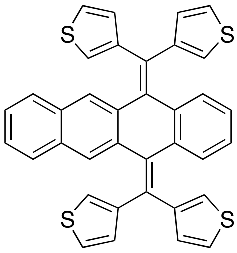

5, 12-bis(dithiophenylmethylene)-5,12-dihydrotetracene (BDPM-DHT)

To a solution of 5,12-bis(dibromomethylene)-5,12-dihydrotetracene (1.13 g, 2.0 mmol) and 3-thiophenyl

boronic acid (2.05 g, 16 mmol) in 50 mL toluene was added 5 ml ethanol and 5 ml water. The mixture was stirred at room temperature and bubbled with nitrogen for 30 minutes. Then K_2CO_3 (2.76 g, 20 mmol) and $Pd(PPh_3)_4$ (0.12 g, 0.1 mmol) were added in one portion and the mixture was heated to reflux for 24 hours. The reaction mixture was cooled to room temperature and filtered through a pad (silicon gel height: 5 cm), washing with dichloromethane until the filtrate was clean. The filtrates were concentrated under reduced pressure and the crude product was obtained. The crude product was then triturated with ethanol and filtered to give product (0.95 g) in 82% yield. Firstly, run fast chromatography using CH_2Cl_2:hexane = 1: 4 as eluent; then dissolve in $CHCl_3$ and put them in the hexane atmosphere, high purity products are obtained as colorless crystals.

^{1}H-NMR($CDCl_3$) δ (ppm): 7.52 (s, 2H), 7.46 (dd, J_1=6.0 Hz, J_2=3.2 Hz, 2H), 7.29-7.17 (m, 10H), 7.12 (dd, J_1=6.0 Hz, J_2=3.2 Hz, 2H), 7.01 (d, J=4.0 Hz, 2H), 6.95 (d, J=4.0 Hz, 2H), 6.89 (dd, J_1=6.4 Hz, J_2=3.2 Hz, 2H).

^{13}C-NMR($CDCl_3$) δ (ppm): 142.02, 141.99, 138.15, 136.00, 135.75, 131.52, 129.27, 129.19, 129.06, 127.93, 127.89, 126.70, 125.93, 125.36, 125.16, 124.31, 124.10.

MALDI-TOF mass: m/z = 582.0591 (cacld. 582.0604).

2. Single crystal structures summary

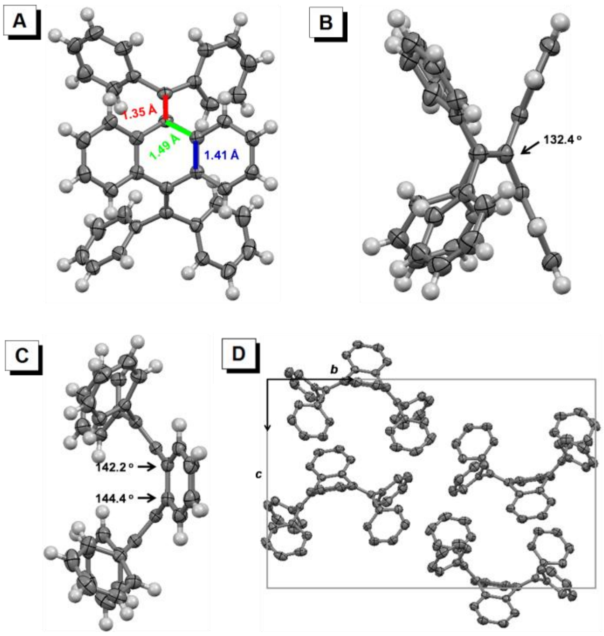

Fig. S1 Crystal structure of BDPM-DHA. (A) Top view and (B)-(C) side view of BDPM-DHA with carbon atom positions shown as 50% probability ellipsoids; (D) molecular packing of BDPM-DHA as viewed along the *a* axis of the unit cell. (Carbon and hydrogen atoms are shown in gray and white, respectively.)

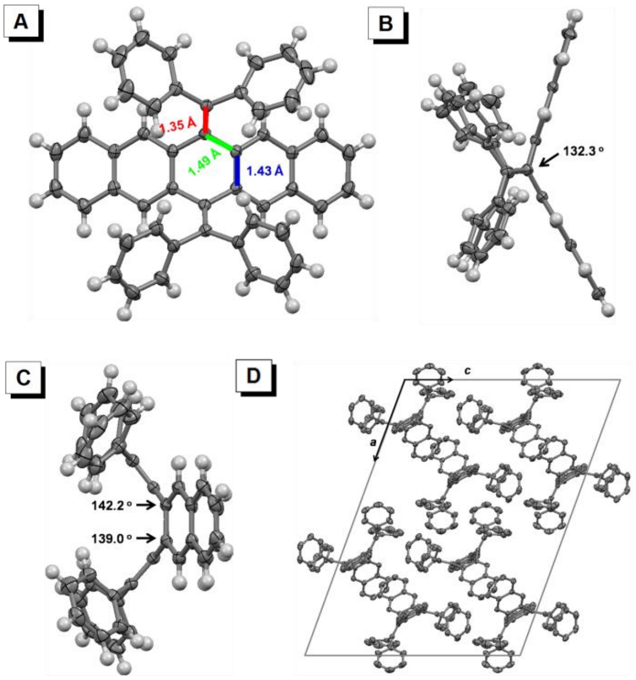

Fig. S2 Crystal structure of BDPM-DHP. (A) Top view and (B)-(C) side view of BDPM-DHP with carbon atom positions shown as 50% probability ellipsoids; (D) molecular packing of BDPM-DHP as viewed along the *b* axis of the unit cell. (Carbon and hydrogen atoms are shown in gray and white, respectively.)

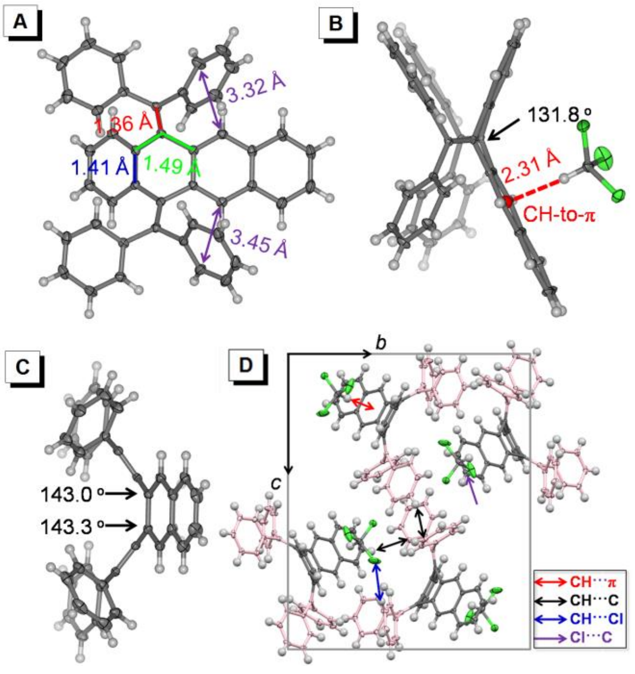

Fig. S3 Crystal structure of BDPM-DHT•$CHCl_3$. (A) Top view and (B)-(C) side view of BDPM-DHT with carbon atom positions shown as 50% probability ellipsoids; (D) molecular packing of BDPM-DHT as viewed along the *a* axis of the unit cell with diphenylmethylene group shown in pink. (Carbon, chlorine and hydrogen atoms are shown in gray, green, and white, respectively.)

Table S1. Crystal data and structure refinement summary

	BDPM-DHA	BDPM-DHT •$CHCl_3$	BDPM-DHT •solvent-free	BDPM-DHP
Empirical formula	$C_{40} H_{28}$	$C_{45} H_{31} Cl_3$	$C_{44} H_{30}$	$C_{48} H_{32}$
Formula weight	508.62	678.05	558.68	608.74
Temperature	173.00(14) K	173.1(5) K	173.00(14) K	173.0(3) K
Wavelength	1.5418 Å	1.5418 Å	1.5418 Å	1.5418 Å
Crystal system	Monoclinic	Monoclinic	Monoclinic	Monoclinic
Space group	P2(1)/c	P2(1)/n	P2(1)	C2/c
Unit cell dimensions	a=9.04620(10)Å b=22.0677(2)Å c=14.11020(10)Å □=90° □=91.1340(10)° □=90°	a=9.6681(2)Å b=17.2389(4)Å c=20.8625(5) Å □= 90° □= 90.222(2)° □ = 90°	a=9.2686(2)Å, b=7.5344(1)Å c=22.0069(5) Å □= 90° □= 91.349(2)° □= 90°	a=28.4000(7) Å, b = 12.0889(2) Å c = 20.7374(6) Å □=90° □=110.271(3)° □=90°
Volume	2816.25(4) $Å^3$	3477.07(14) $Å^3$	1536.39(5) $Å^3$	6678.7(3) $Å^3$
Z	4	4	2	8
Density (calculated)	1.200 Mg/m^3	1.295 Mg/m^3	1.208Mg/m^3	1.211 Mg/m^3
Absorption coefficient	0.513 mm^{-1}	2.621 mm^{-1}	0.517 mm^{-1}	0.519 mm^{-1}
F(000)	1072	1408	588	2560
Theta range for data collection	9.65 to 66.98°	3.33 to 66.99°	6.21 to 71.64°	4.02 to 66.98°
Index ranges	-10<=h<=10, -26<=k<=25, -16<=l<=16	-10<=h<=11, -20<=k<=20, -24<=l<=24	-11<=h<=11, -9<=k<=6, -26<=l<=26	33<=h<=33, -11<=k<=14, -24<=l<=22
Reflections collected	26331	16573	8918	19414
Independent reflections	4938 [R(int) = 0.0300]	5865 [R(int) = 0.0705]	4245 [R(int) = 0.0337]	5903 [R(int) = 0.0349]
Completeness to theta = 66.50°	99.34 %	96.50 %	99.44 %	99.34 %
Max. and min. transmission	1.00000 and 0.32440	1.00000 and 0.52865	1.00000 and 0.33995	1.00000 and 0.80462
Data / restraints / parameters	4938 / 0 / 361	5865 / 6 / 461	4245 / 1 / 397	5903 / 0 / 433
Goodness-of-fit on F^2	1.005	1.002	1.005	1.002
Final R indices [I>2sigma(I)]	R1 = 0.0383, wR2 = 0.0911	R1 = 0.0779, wR2 = 0.1766	R1 = 0.0557, wR2 = 0.1504	R1 = 0.0383, wR2 = 0.0946
R indices (all data)	R1 = 0.0407, wR2 = 0.0927	R1 = 0.0876, wR2 = 0.1815	R1 = 0.0578, wR2 = 0.1517	R1 = 0.0409, wR2 = 0.0966
Largest diff. peak and hole	0.143 and -0.140 e.$Å^{-3}$	0.348 and -0.340 e.$Å^{-3}$	0.352 and -0.246 e.$Å^{-3}$	0.166 and -0.166 e.$Å^{-3}$

Table S2. Crystal structure analysis of possible reactive position.

	BDPM-DHA (solvent free)	BDPM-DHP (solvent free)	BDPM-DHT •$CHCl_3$	BDPM-DHT (solvent free)
Distance 1 (Å)	3.456 /3.513	3.304 /3.870	3.313 /3.473	3.274 /3.609
Distance 2 (Å)	3.335 /4.001	3.538 /3.574	3.455 /3.532	3.459 /3.578
Distance 3 (Å)	3.789 /3.323	3.329 /3.416	3.144 /4.135	3.071 /3.472
Distance 4 (Å)	3.265 /3.939	3.584 /3.689	3.203 /3.891	3.411 /3.802

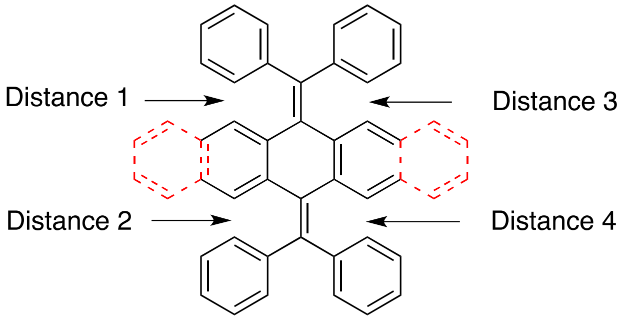

3. Aggregation-induced emission properties of BDPM-DHA and BDPM-DHP

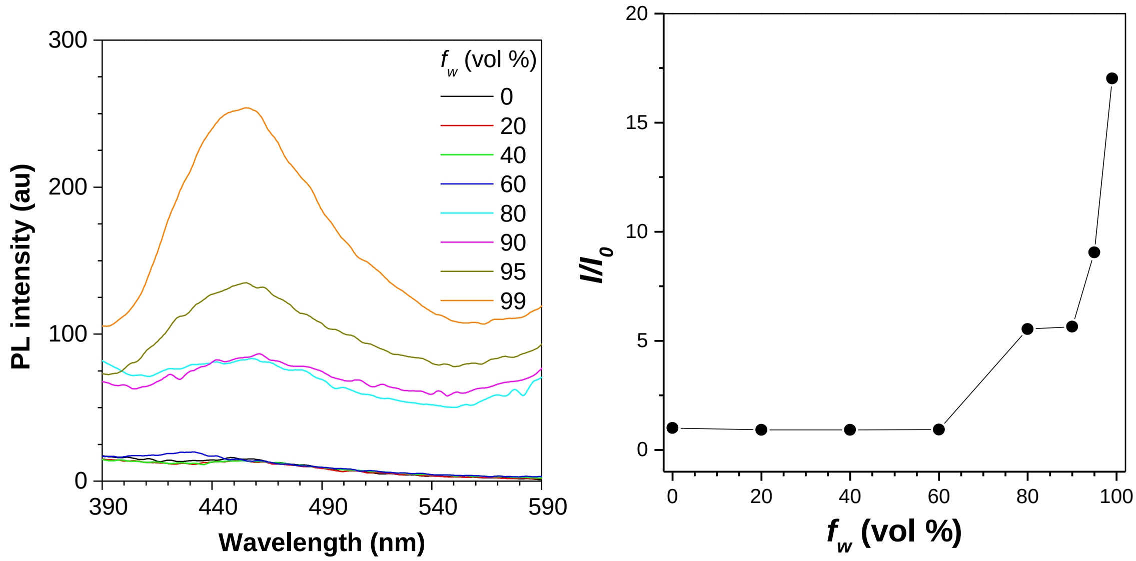

Fig. S4 (Left) PL spectra of BDPM-DHA in THF/water mixtures with different water fractions (f_w). (Right) Plot of PL peak intensity *vs* water fraction (f_w) of the aqueous mixture. (Concentration: 10 µM; excitation wavelength: 320 nm.)

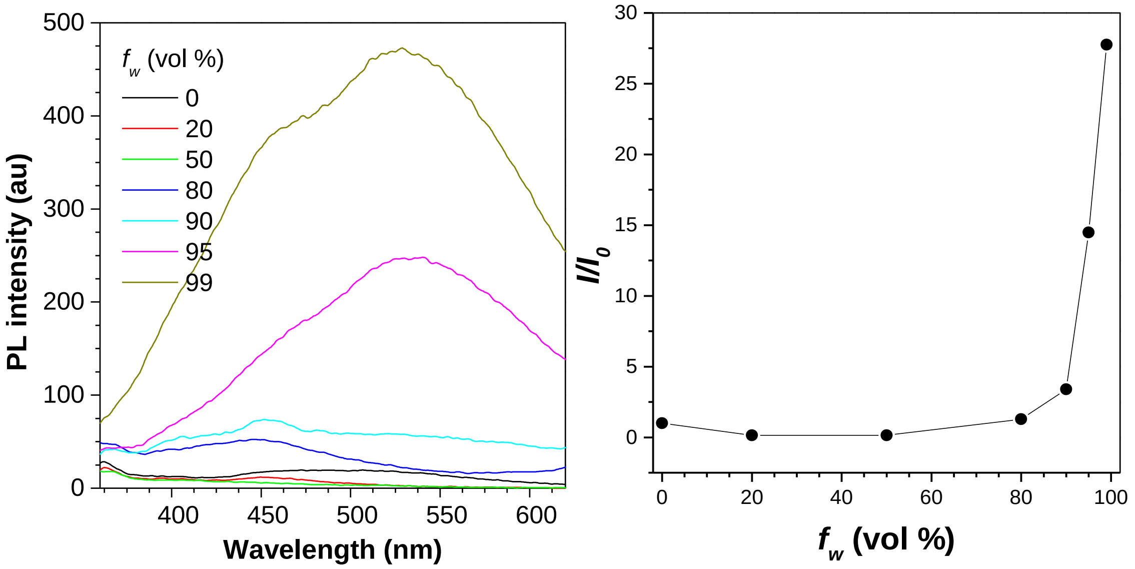

Fig. S5 (Left) PL spectra of BDPM-DHP in THF/water mixtures with different water fractions (f_w). (Right) Plot of PL peak intensity *vs* water fraction (f_w) of the aqueous mixture. (Concentration: 10 μM; excitation wavelength: 340 nm.)

4. UV-vis absorption in solution

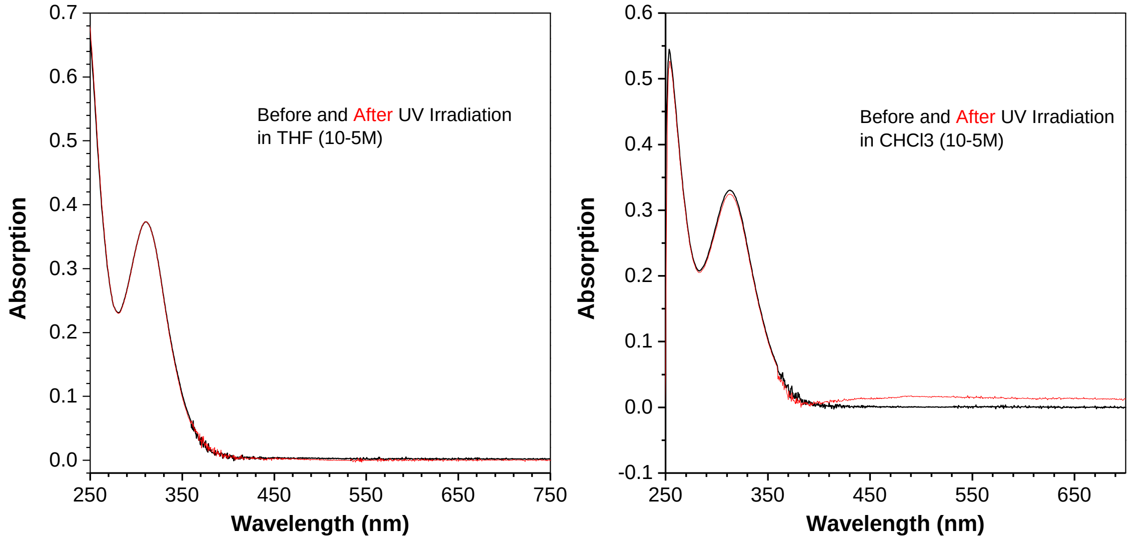

Fig. S6 UV-vis absorption spectra of BDPM-DHT in THF (left) and chloroform (right) solutions before (dark line) and after (red line) UV lamp shining for 5 minutes.

5. UV-vis reflectance absorption and PL

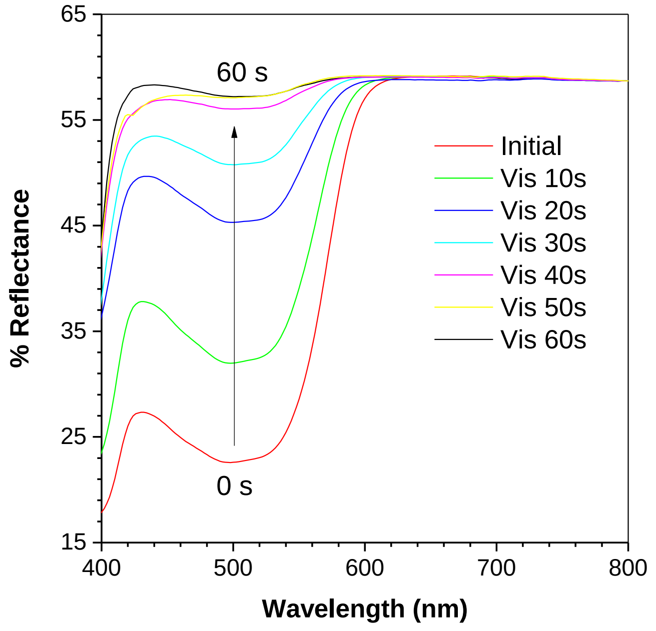

Fig. S7 Changes in the UV-vis reflectance spectra of BDPM-DHT crystal upon room light treatment (Initial: UV irradiation at λ=365 nm for 3 minutes).

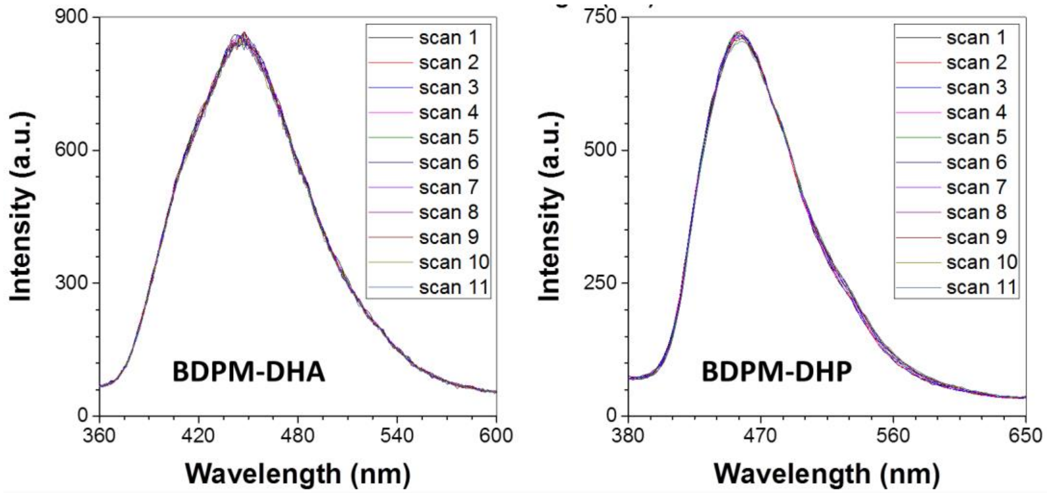

Fig. S8 PL spectra of BDPM-DHA and BDPM-DHP film (grinding their crystals by quartz plates) with multiple scanning (excitation wavelength: 340 nm.)

6. Differential scanning calorimetry and thermal gravimetric analysis

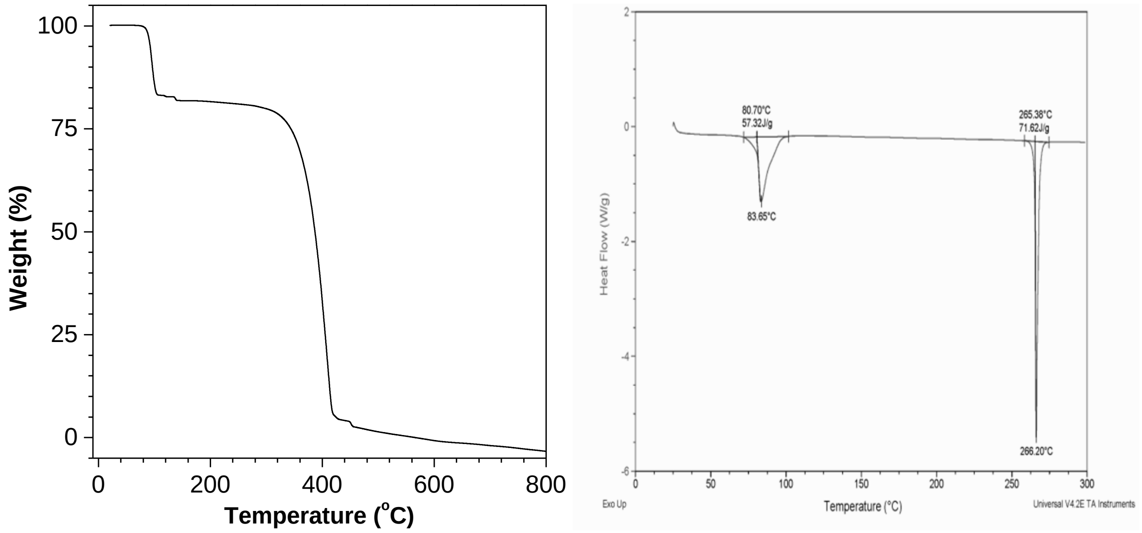

Fig. S9 TGA and DSC thermogram of BDPM-DHT at a heating rate of 10 °C/min. (One $CHCl_3$ molecule per BDPM-DHT molecule was trapped in the crystal lattice and can be removed at ~80 degree with crystal collapse)

7. Instruments for *in-situ* single crystal X-ray analysis

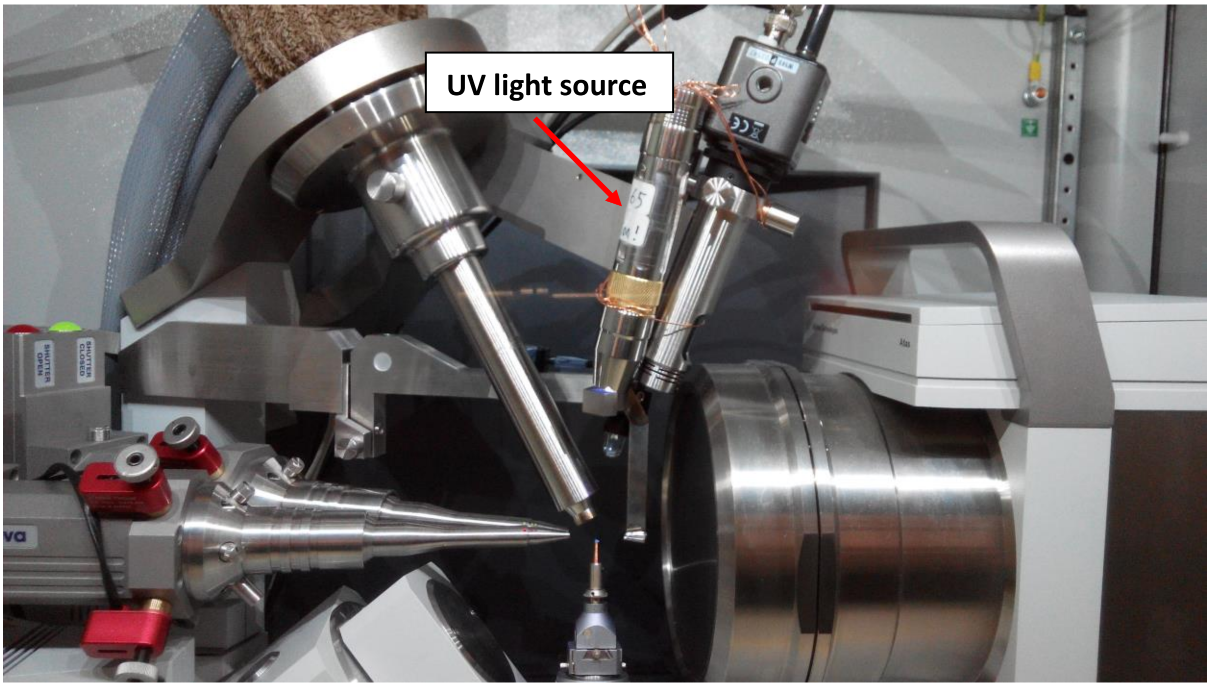

Fig. S10 Instrument for collecting X-ray crystallography data with continuous UV irradiation.

8. Photocyclization of *cis*-stillbene

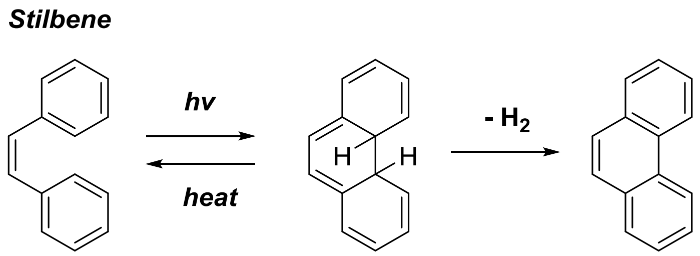

Scheme S2. Photocyclization of *cis*-stillbene and its oxidation to phenanthrene.

9. Density functional theory calculation

The systems were studied by the DFT calculations performed with the Gaussian 09 program.[1] The geometries were first optimized with B3LYP hybrid density function with the 6-31G (d,p) basis set. The single-point energy and the energy levels of HOMO and LUMO were calculated with the 6-311++G (d,p) basis set.

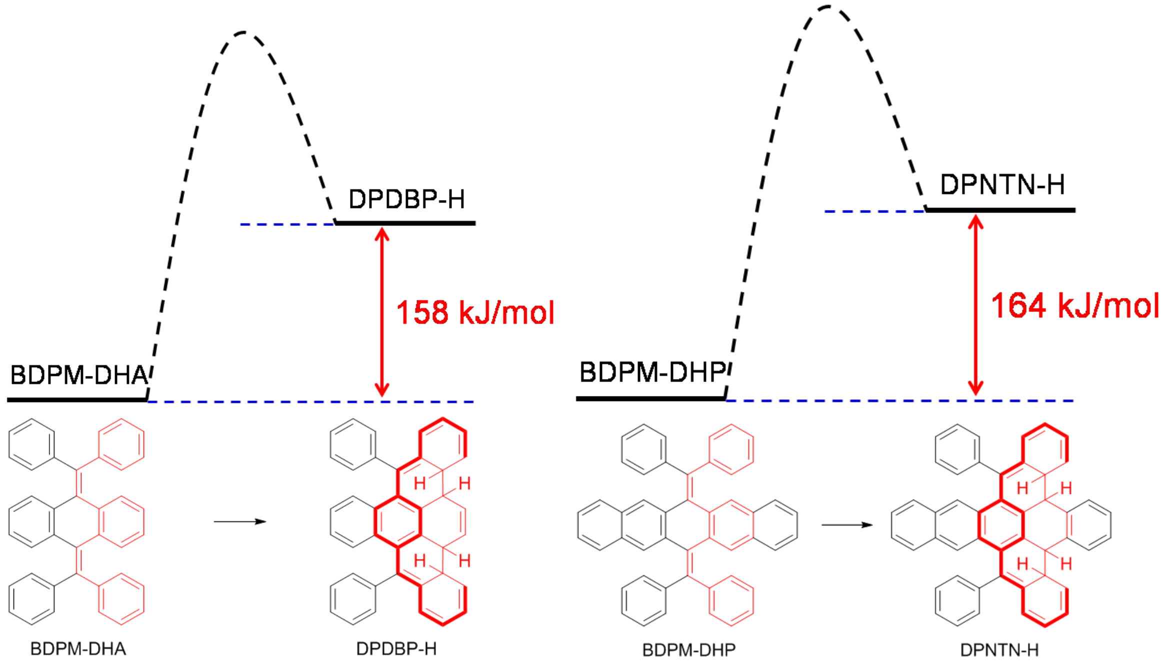

Fig. S11 Calculated energy differences of BDPM-DHA system (left) and BDPM-DHP system (right).

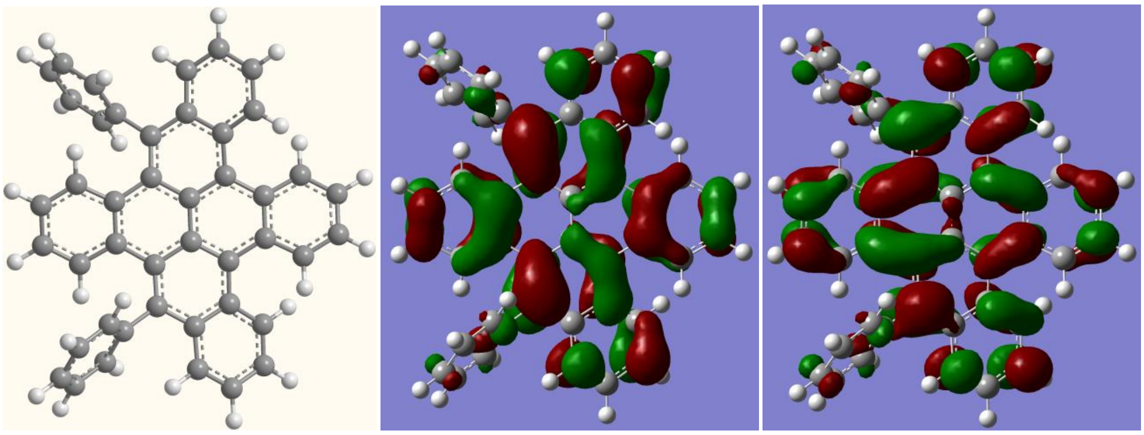

Molecular Structure HOMO = −5.385 eV LUMO = −2.171 eV

Fig. S12 Calculated molecular structure (left) and molecular orbital amplitude plots (isovalue = 0.02) of HOMO (middle) and LUMO (right) energy levels of DPBNP.

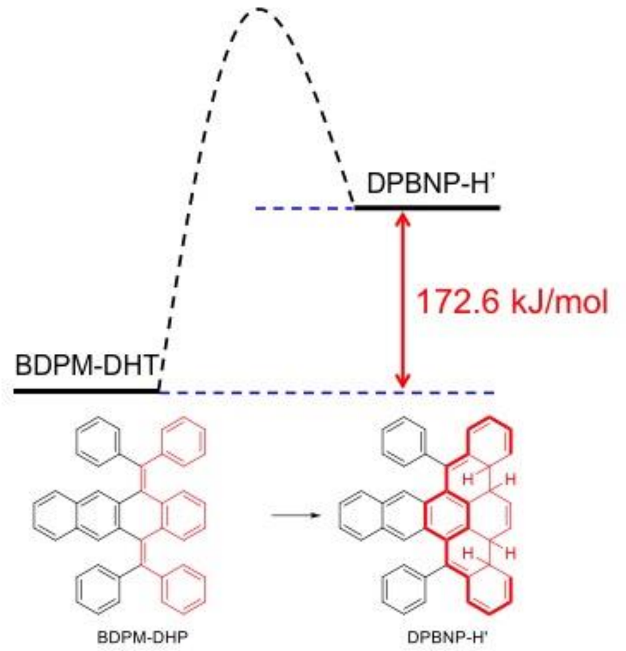

Fig. S13 Calculated energy differences of BDPM-DHT and DPBNP-H'.

10. High resolution mass spectrum

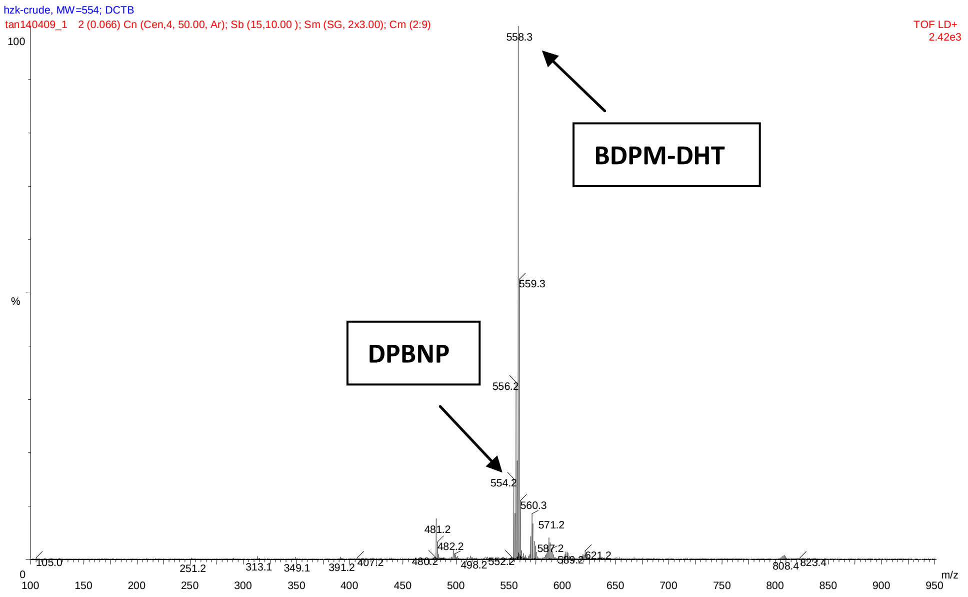

Fig. S14 High resolution mass spectrum of BDPM-DHT crystals exposed to UV light for two days in air.

11. ^{1}H-NMR analysis of photocyclization mechanism

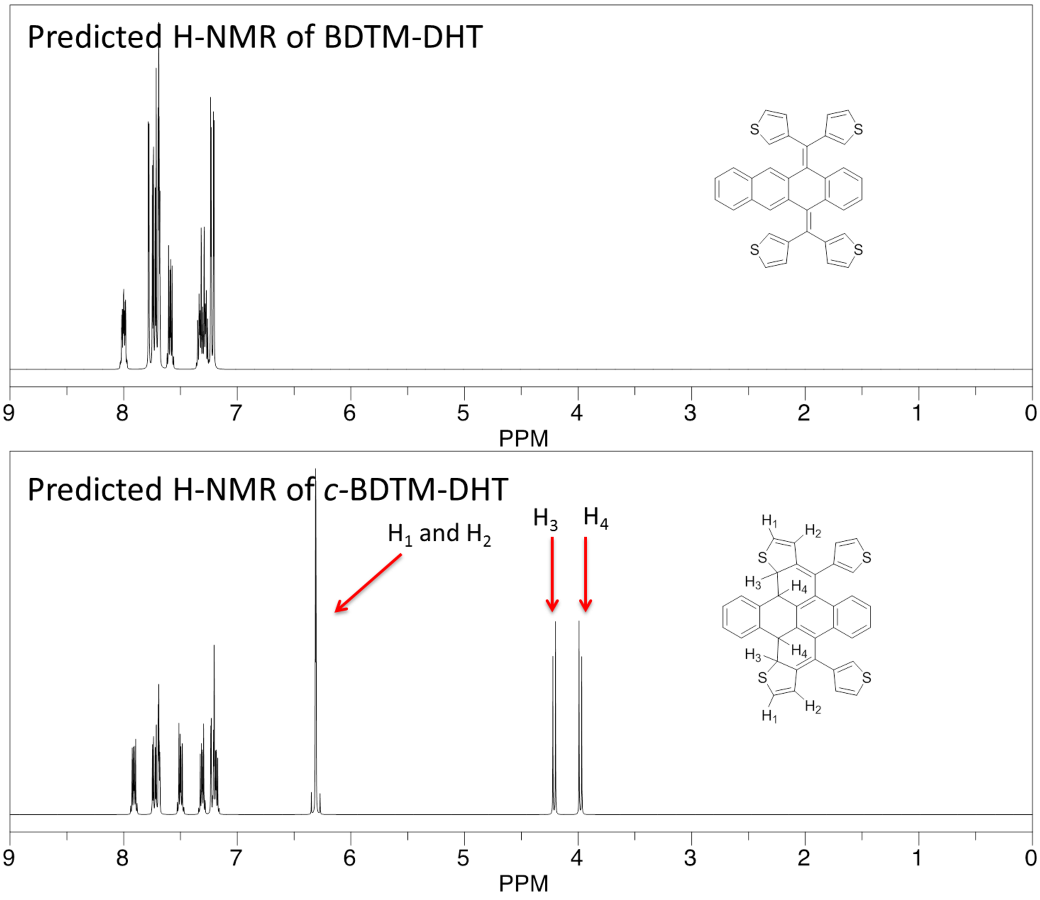

Fig. S15 Predicted H-NMR spectra of BDTM-DHT and *c*-BDTM-DHT by ChemDraw.

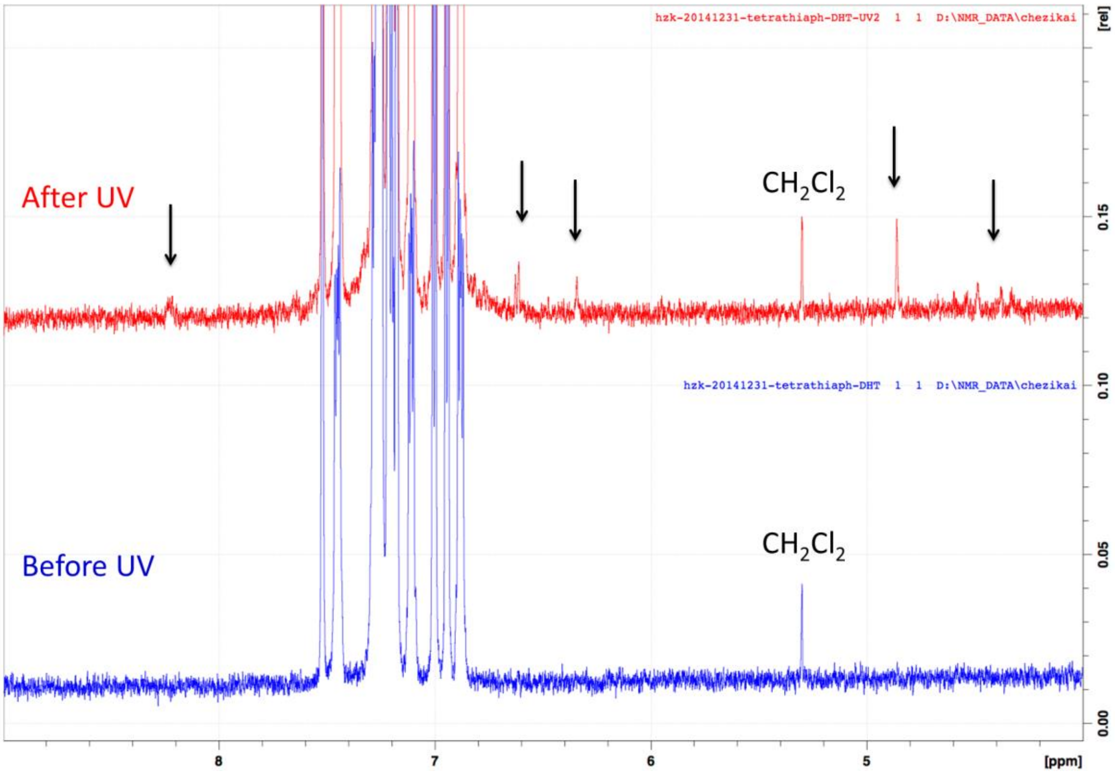

Fig. S16 H-NMR spectra of BDTM-DHT (before (down) and after (up) UV irradiation) in $CDCl_3$.

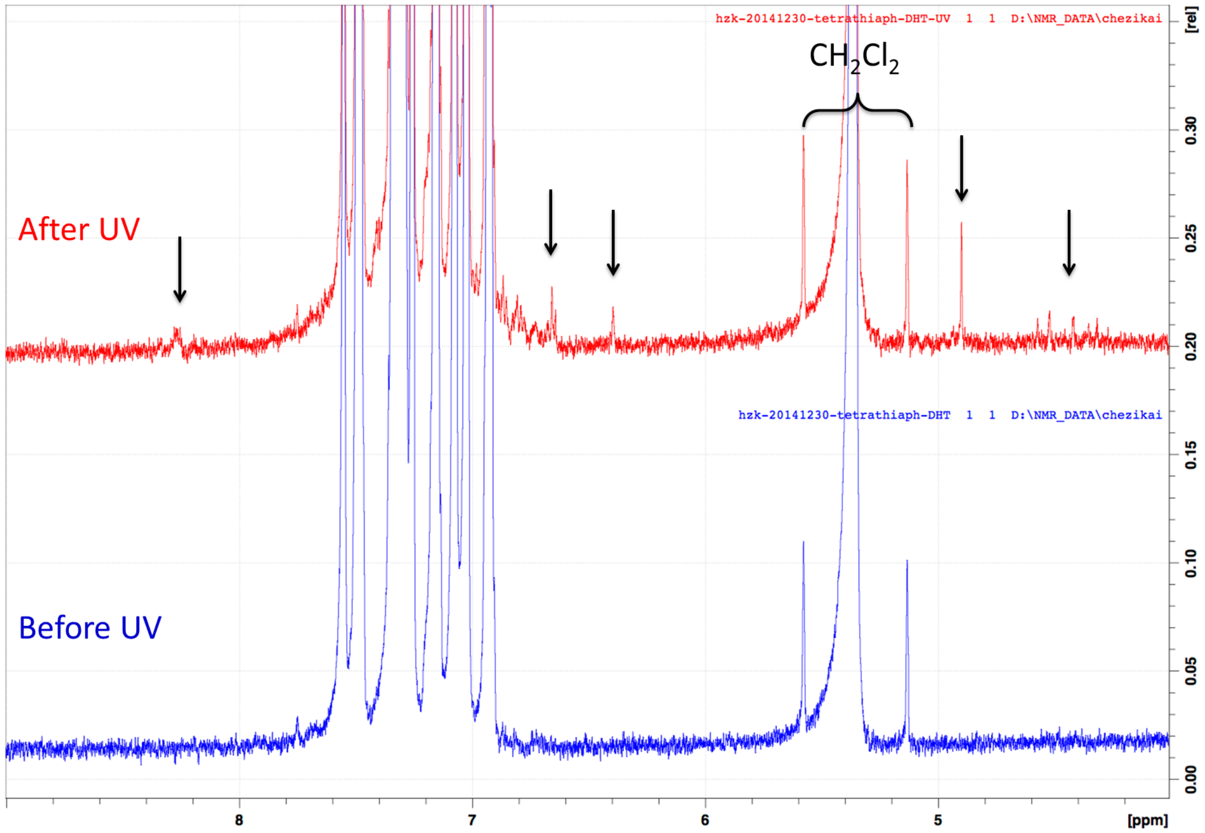

Fig. S17 H-NMR spectra of BDTM-DHT (before (down) and after (up) UV irradiation) in CD_2Cl_2.

12. Electronic structures summary

Table S3 Relative energy[a], LUMO−HOMO gap, absorption edge and energy levels of LUMO and HOMO for BDPM-DHA, BDPM-DHT and BDPM-DHP.

Compound	BDPM-DHA	BDPM-DHT	BDPM-DHP
Relative energy (kJ/mol)[b]	0	149	43 (+2 $\mathbf{H_2}$)
LUMO (eV)[b]	−1.70	−2.47	−2.17
HOMO (eV)[b]	−5.79	−4.87	−5.38
$Gap^{Cal.}$ (eV)[b]	4.09	2.40	3.21
$Gap^{Opt.}$ (eV)[c]	3.63	2.09	3.08
Absorption edge (nm)	342	594	403

[a] The energy of BDPM-DHA is set as 0. [b] The single-point energy and the energy levels of HOMO and LUMO was optimized with the 6-31G(d,p) basis set, and calculated with the 6-311++G(d,p) basis set. [c] Optical Gap=1240/λ, (λ: absorption edge).

13. NMR spectra

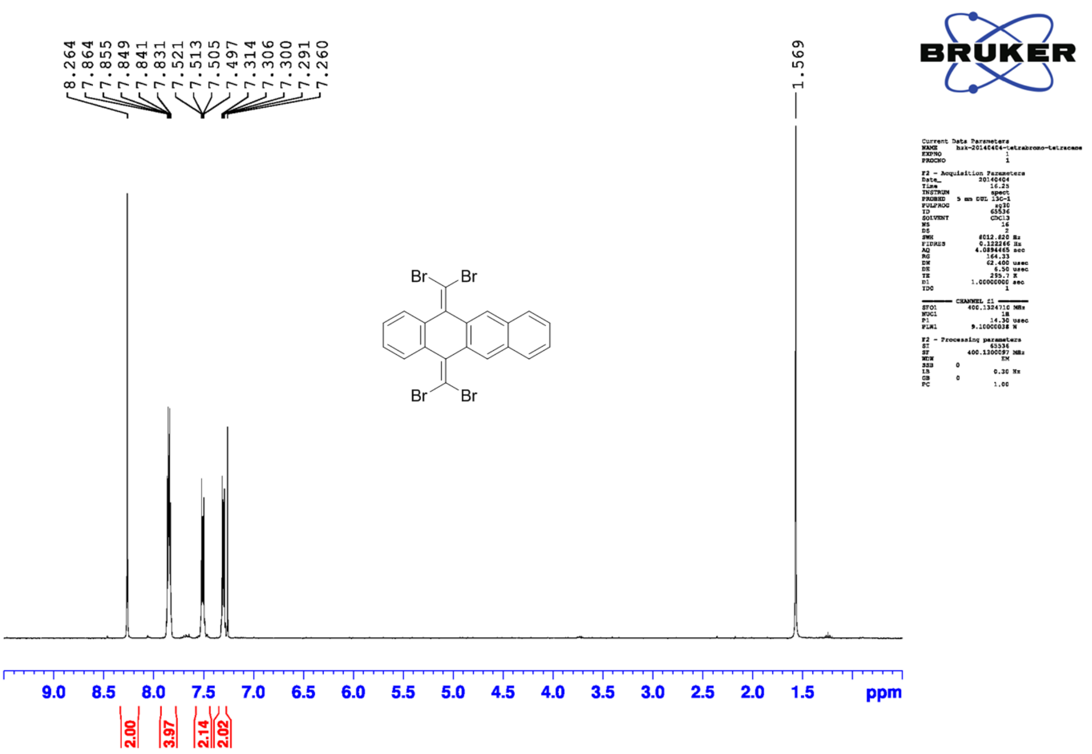

^{1}H-NMR of 5, 12-bis(dibromomethylene)-5,12-dihydrotetracene in $CDCl_3$.

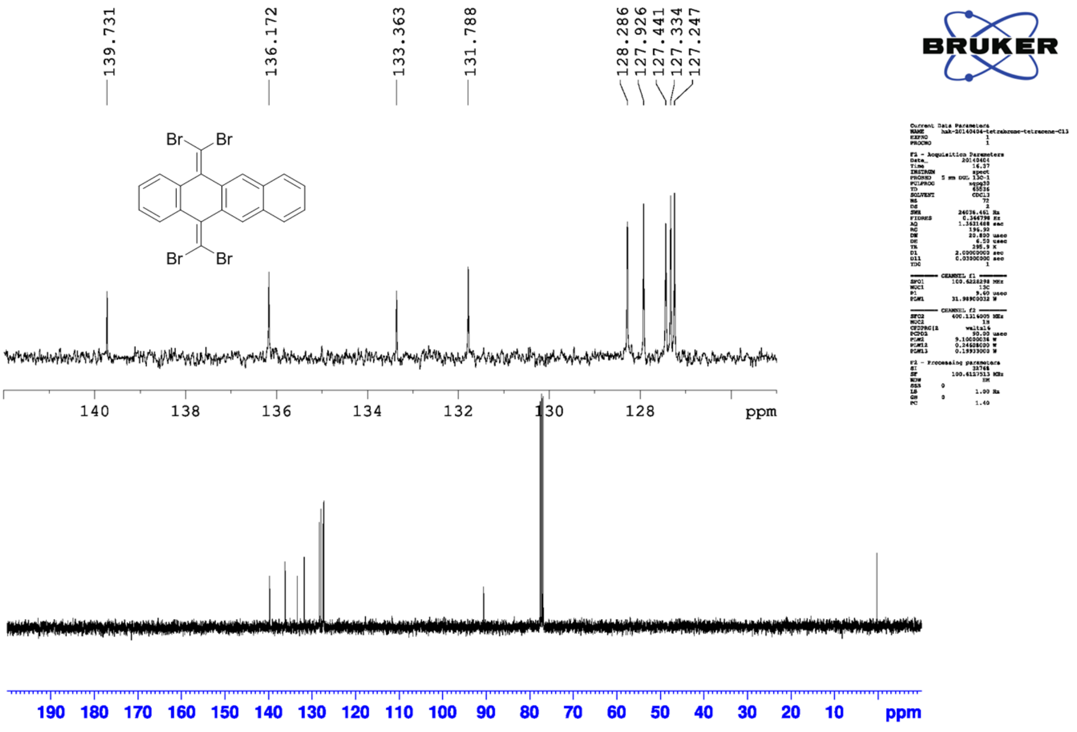

^{13}C-NMR of 5, 12-bis(dibromomethylene)-5,12-dihydrotetracene in $CDCl_3$.

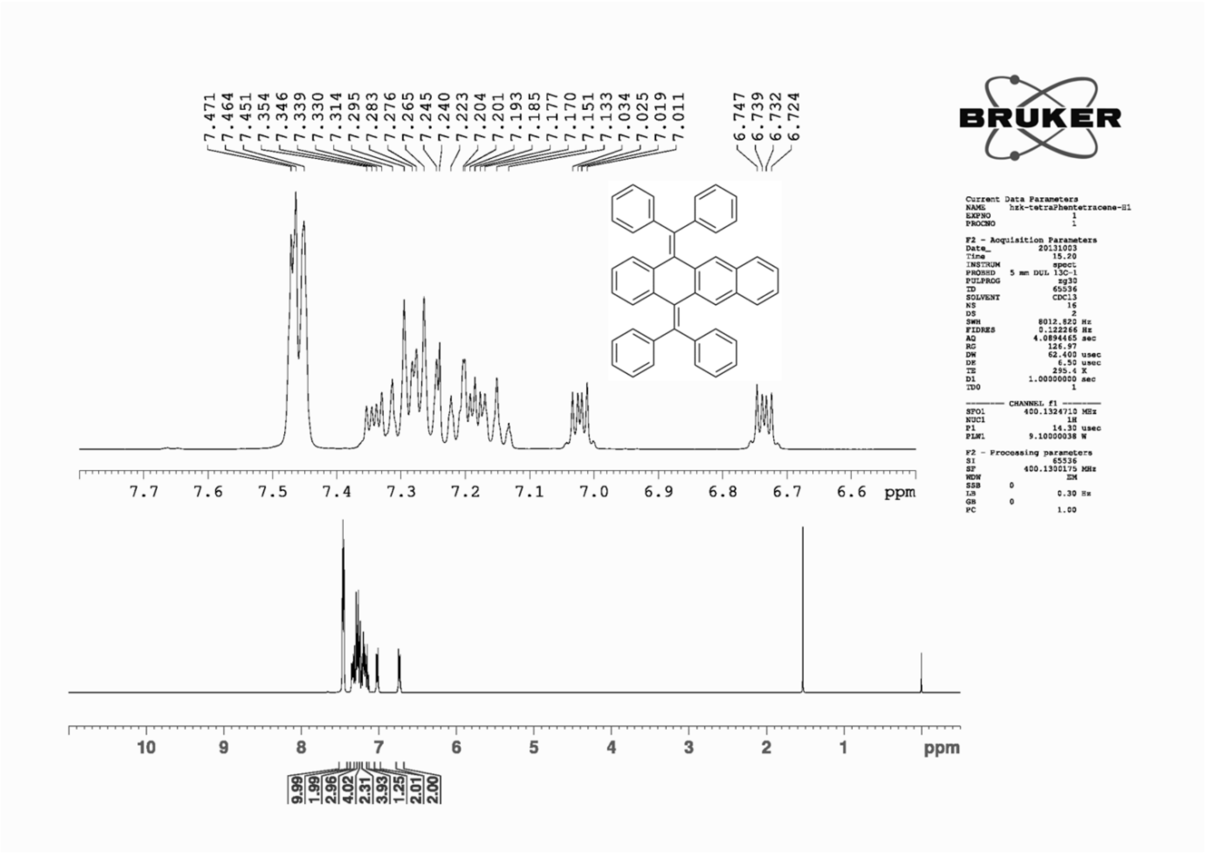

^{1}H-NMR of 5, 12-bis(diphenylmethylene)-5,12-dihydrotetracene in $CDCl_3$.

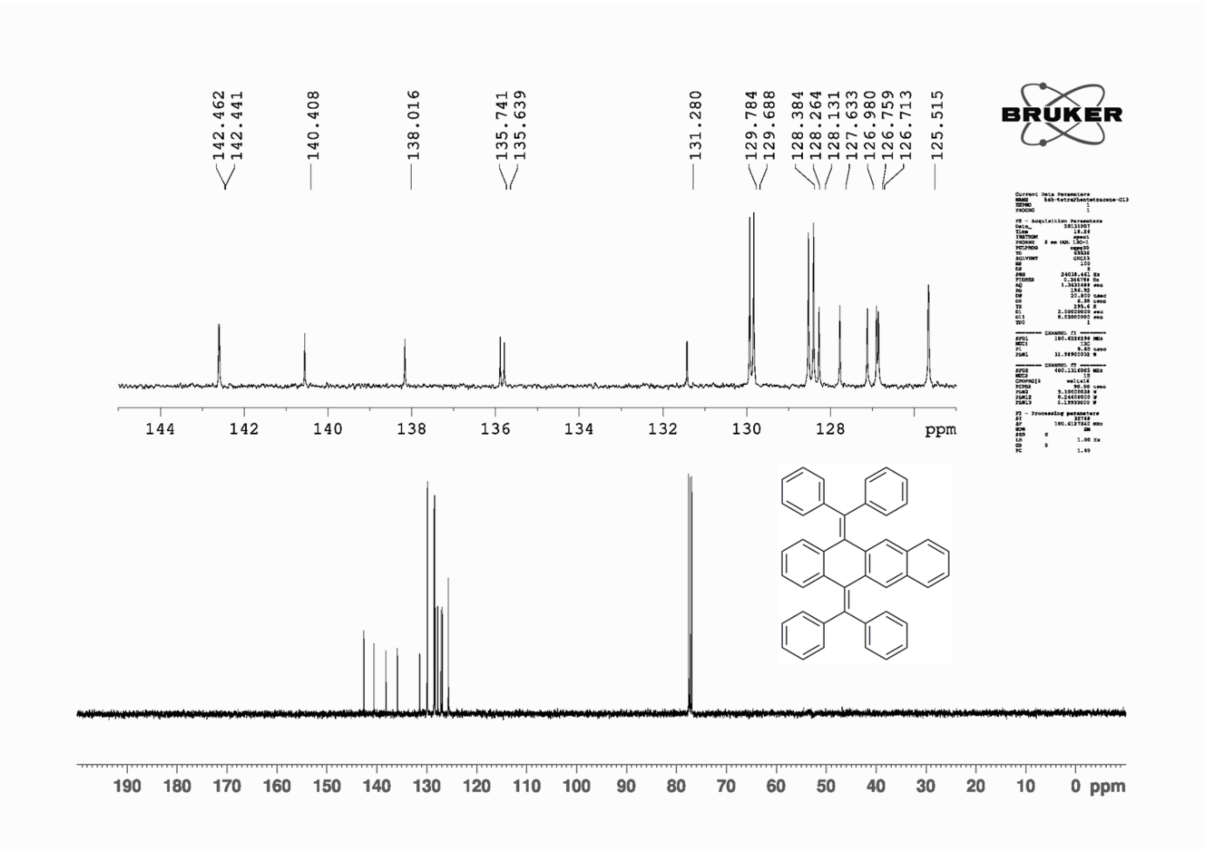

^{13}C-NMR of 5, 12-bis(diphenylmethylene)-5,12-dihydrotetracene in $CDCl_3$.

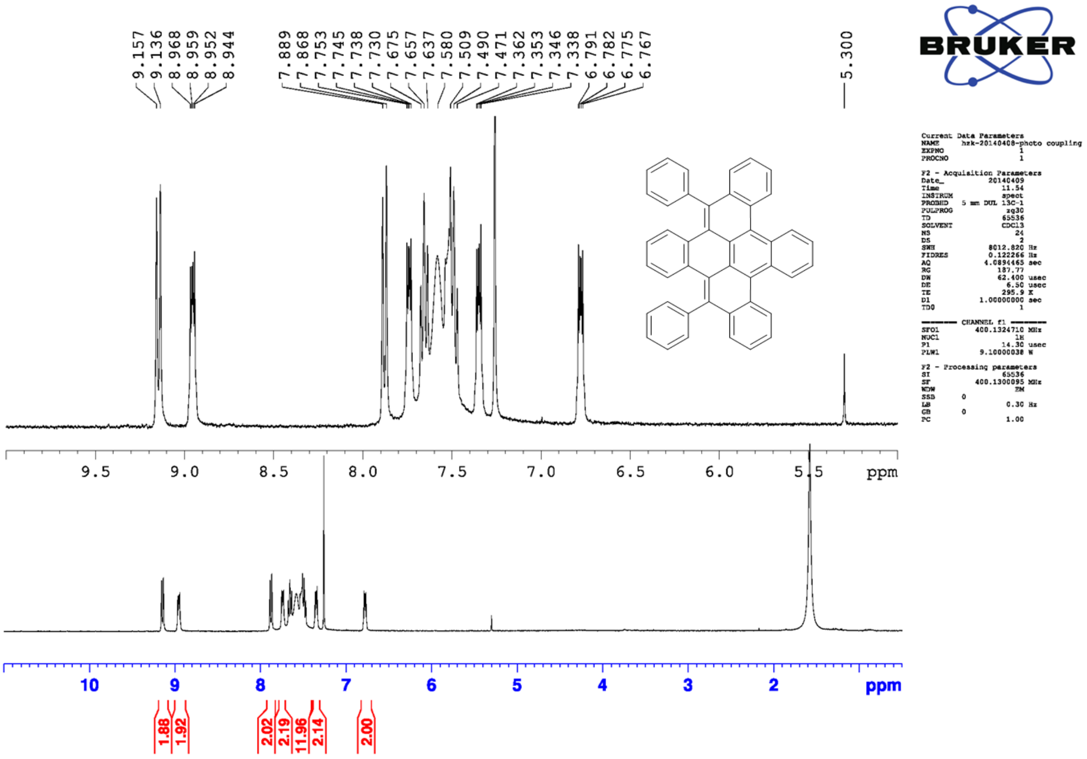

^{1}H-NMR of 13,18-diphenylbenzo[*h*]naphtho[*1*,*2*,*3*,*4*-*rst*]pentaphene in $CDCl_3$.

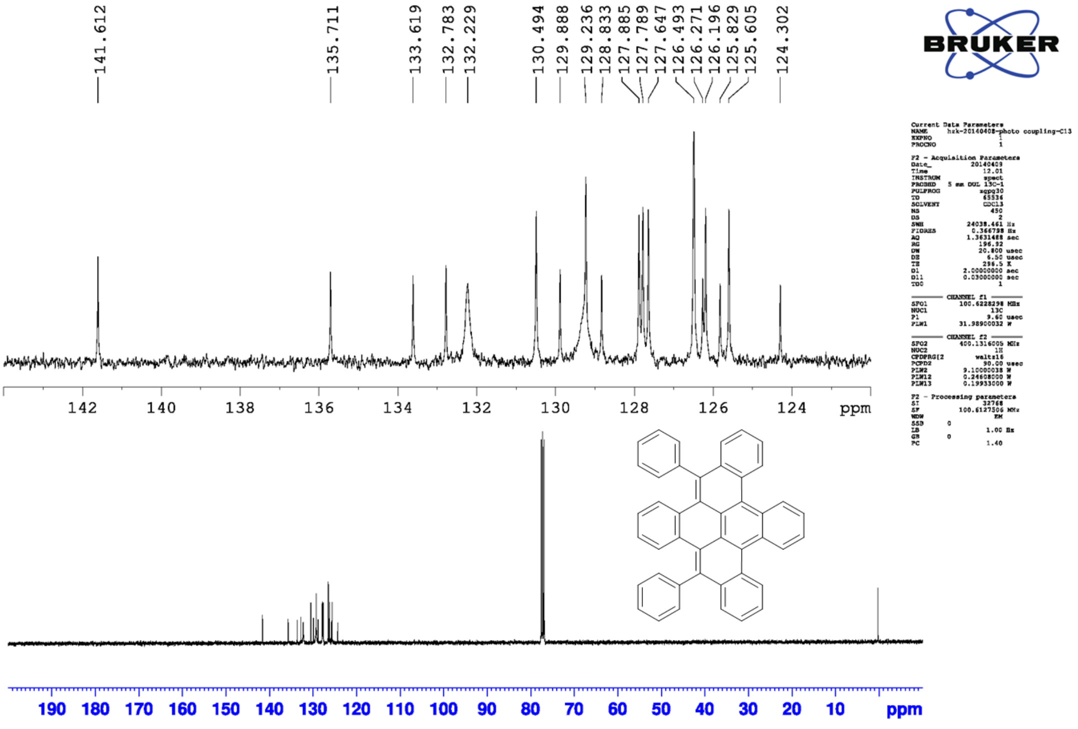

^{13}C-NMR of 13,18-diphenylbenzo[*h*]naphtho[*1*,*2*,*3*,*4*-*rst*]pentaphene in $CDCl_3$.

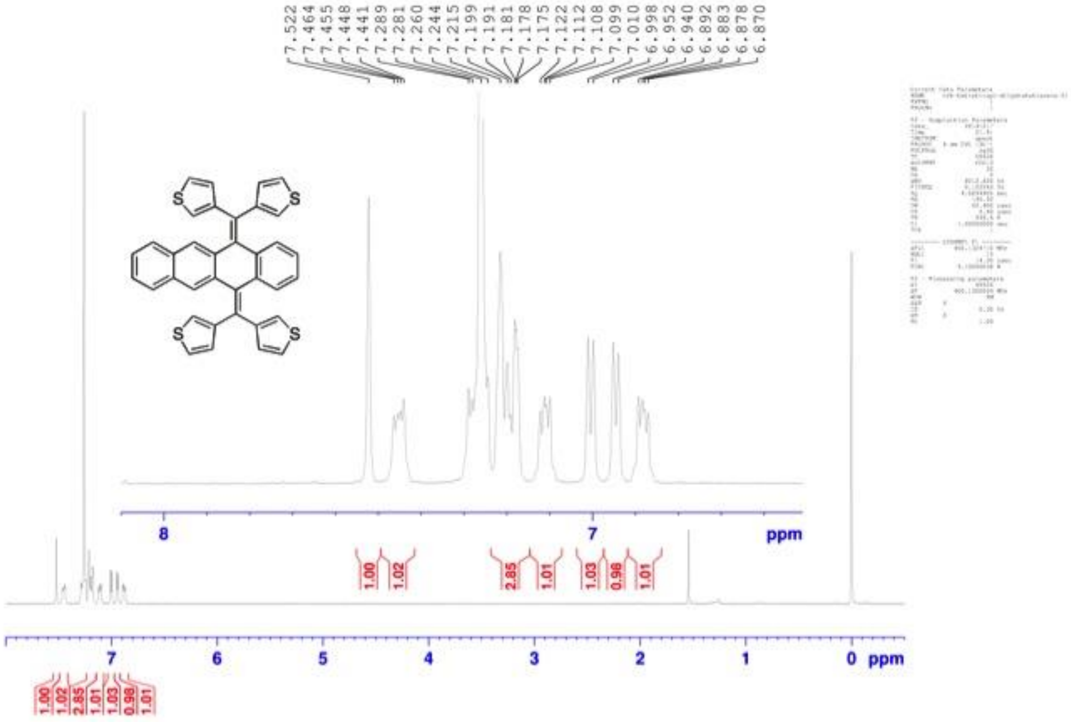

^{1}H-NMR of 5, 12-bis(dithiophenylmethylene)-5,12-dihydrotetracene in $CDCl_3$.

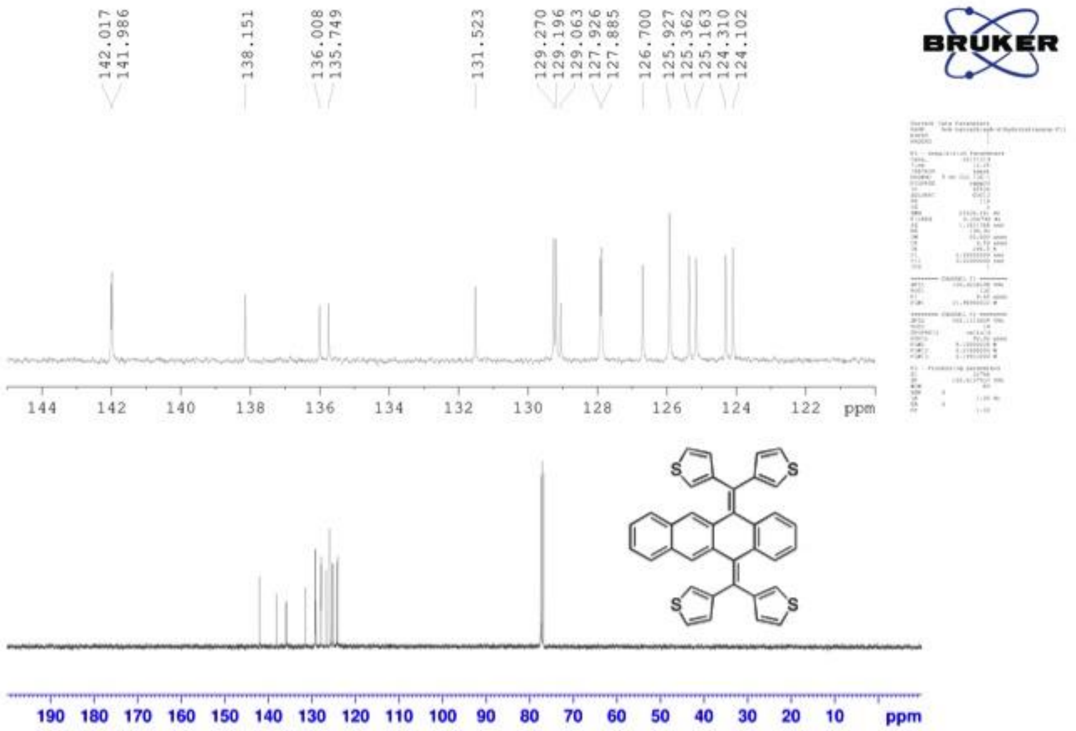

^{13}C-NMR of 5, 12-bis(dithiophenylmethylene)-5,12-dihydrotetracene in $CDCl_3$.

(1) Gaussian 09, Revision A.1, M. J. Frisch, G. W. Trucks, H. B. Schlegel, G. E. Scuseria, M. A. Robb, J. R. Cheeseman, G. Scalmani, V. Barone, B. Mennucci, G. A. Petersson, H. Nakatsuji, M. Caricato, X. Li, H. P. Hratchian, A. F. Izmaylov, J. Bloino, G. Zheng, J. L. Sonnenberg, M. Hada, M. Ehara, K. Toyota, R. Fukuda, J. Hasegawa, M. Ishida, T. Nakajima, Y. Honda, O. Kitao, H. Nakai, T. Vreven, J. A. Montgomery, Jr., J. E. Peralta, F. Ogliaro, M. Bearpark, J. J. Heyd, E. Brothers, K. N. Kudin, V. N. Staroverov, R. Kobayashi, J. Normand, K. Raghavachari, A. Rendell, J. C. Burant, S. S. Iyengar, J. Tomasi, M. Cossi, N. Rega, J. M. Millam, M. Klene, J. E. Knox, J. B. Cross, V. Bakken, C. Adamo, J. Jaramillo, R. Gomperts, R. E. Stratmann, O. Yazyev, A. J. Austin, R. Cammi, C. Pomelli, J. W. Ochterski, R. L. Martin, K. Morokuma, V. G. Zakrzewski, G. A. Voth, P. Salvador, J. J. Dannenberg, S. Dapprich, A. D. Daniels, Ö. Farkas, J. B. Foresman, J. V. Ortiz, J. Cioslowski, and D. J. Fox, Gaussian, Inc., Wallingford CT, 2009.
